# A pyroptosis-related gene model and its correlation with the microenvironment of lung adenocarcinoma: A bioinformatics analysis and experimental verification

**DOI:** 10.3389/fgene.2022.997319

**Published:** 2022-11-09

**Authors:** Yi Dong, Lina Yi, Qibin Song, Yi Yao

**Affiliations:** ^1^ Cancer Center, Renmin Hospital of Wuhan University, Wuhan, China; ^2^ Hubei Provincial Research Center for Precision Medicine of Cancer, Wuhan, China

**Keywords:** pyroptosis, cancer, immunity, microenvironment, NLRC4, bioinformatics

## Abstract

**Background:** Non-small cell lung cancer, comprising lung adenocarcinoma (LUAD) and lung squamous cell carcinoma, is one of the leading causes of cancer-related mortality. Pyroptosis is a new form of programmed cell death involved in cancer development. The relationship between LUAD and pyroptosis is unclear. This research aims to investigate this relationship and develop a stratified clinical model based on pyroptosis-related genes (PRGs).

**Methods:** We analyzed the data of LUAD from The Cancer Genome Atlas (TCGA) and evaluated the expression of 48 PRGs to identify the differentially expressed genes. Then, constructing the risk model using the least absolute shrinkage and selection operator and the Cox regression method to find the gene signatures. The functional enrichment, immune cell infiltration, tumor mutational burden (TMB), and expression of immune checkpoints were compared to investigate the potential mechanism. The IC50 of common drugs was evaluated and compared. The inflammasome activation assay and lactate dehydrogenase (LDH) assay of NLR-family CARD-containing protein 4 (NLRC4) were also performed to confirm the role of pyroptosis in LUAD.

**Results:** The pyroptosis-related model accurately predicted the prognosis of patients with LUAD, with the low-risk group exhibiting a higher survival probability. The risk score was an independent prognostic factor for survival. The stratified patients exhibited distinct tumor microenvironments, TMB, and drug sensitivity. The validation experiments of NLRC4 confirmed its role in inducing pyroptosis *via* promoting IL-1 maturation.

**Conclusion:** PRGs regulated the tumor microenvironment and influenced the outcome of LUAD. NLRC4 may function as a hub gene in the process of LUAD.

## Introduction

Lung cancer is the leading cause of cancer-related mortality worldwide, with 1.8 million newly diagnosed cases and 1.6 million deaths per year (Bray, et al., 2018). More than 85% of patients with lung cancer are diagnosed with non-small cell lung cancer (NSCLC), with lung adenocarcinoma (LUAD) and lung squamous cell carcinoma being the most common subtypes (Duma, et al., 2019). Numerous studies have been conducted on the strategies against lung cancer, leading to a significant decrease in mortality and an increase in survival due to advances in diagnosis and treatment. For those tissue biopsies might not be technically feasible can take liquid biopsies, detect ctDNA in plasma, and obtain molecular information (Thai, et al., 2021). With the advent of novel technologies such as next-generation sequencing, lung cancer treatment has entered the molecular era, the development of target therapy and immunotherapy have marked a turning point in cancer treatment. Early clinical trials with these therapies revealed rapid and long-lasting responses in 14%–20% of patients with pretreated advanced NSCLC (Wu, et al., 2020). Although great progress has been made in this field, significant obstacles remain: the mechanism of target therapy resistance; the treatment for rare somatic activating oncogene mutations; as well as the biomarkers to predict the response of anti-cancer therapy (Hirsch, et al., 2017). During the past decade, although the discovery of predictive biomarkers has created new therapeutic opportunities with targeted therapy and immunotherapy, there are still many limitations. Programmed death-ligand 1 (PD-L1) is a predictive biomarker used to guide treatment decisions for its expression is associated with an increased likelihood of response to programmed death-1 (PD-1) pathway blockade, but responses to immune checkpoint inhibitors (ICIs) can also be seen in patients with no tumor PD-L1 expression. High tumor mutational burden (TMB) might be predictive of response to ICIs without any prospective validation. Moreover, the mutation of on- and off-target resistance has not been solved successfully, which needs further investigation for better clinical practice (Thai, et al., 2021). Thus, new biomarkers need to be urgently discovered to learn more about the pathogenesis of LUAD so that new targets can be developed.

According to the prevailing opinion, the 10 hallmarks of cancer lead to cancer initiation and progression (Hanahan and Weinberg, 2011). Among these, the ability to resist cell death and escape immunological damage was discussed in this study. Cell death is a physiological process that regulates cell proliferation, stress response, and immunological response, as well as inhibits tumor growth. Besides apoptosis and necrosis, autophagy, anoikis, and pyroptosis were also described (Fernandes-Alnemri, et al., 2007). Pyroptosis is an inflammatory form of programmed cell death initiated by caspase 1/4/5/11. It is triggered by certain inflammasomes and results in cell swelling, plasma membrane lysis, chromatin fragmentation, and releasing of intracellular proinflammatory components (Fang, et al., 2020). Recent scientific advances have led to the identification of numerous genes as essential regulators of pyroptosis. The relationship between cancer and pyroptosis is intricate. Paclitaxel and cisplatin triggered pyroptosis in A549 *via* the caspase 3/gasdermin E (GSDME) pathway; the effectiveness was associated with the expression of GSDME (Zhang, et al., 2019). However, another study revealed that GSDME was associated with radioresistant lung cancer cells, and its expression was indicative of a poor prognosis for LUAD (Wei, et al., 2020). Apart from its prognostic value, pyroptosis increases the immunological defenses of the host and contributes to the release of tumor antigens, a previous study demonstrated that pyroptosis stimulated the activation of CD8^+^ T lymphocytes and inhibited tumor growth and migration (Tang, et al., 2020).

Available evidence indicates that pyroptosis plays an essential but complex role in tumors. Less attention has been paid to its precise mechanism in LUAD, particularly the impact of the hub genes on the microenvironment and anti-cancer immunity. With new technology, the appropriate treatment based on the gene expression pattern to optimize therapeutic benefits has developed. In this study, we examined the expression pattern of pyroptosis-related genes (PRGs) in LUAD, assessed their clinical utility, investigated the relationship between pyroptosis and TME, and provided therapeutic suggestions. We also explored the role of NLR-family CARD-containing protein 4 (NLRC4) in LUAD to find a new biomarker or therapeutic target.

## Materials and methods

### Dataset collection and processing

**FIGURE 1:**
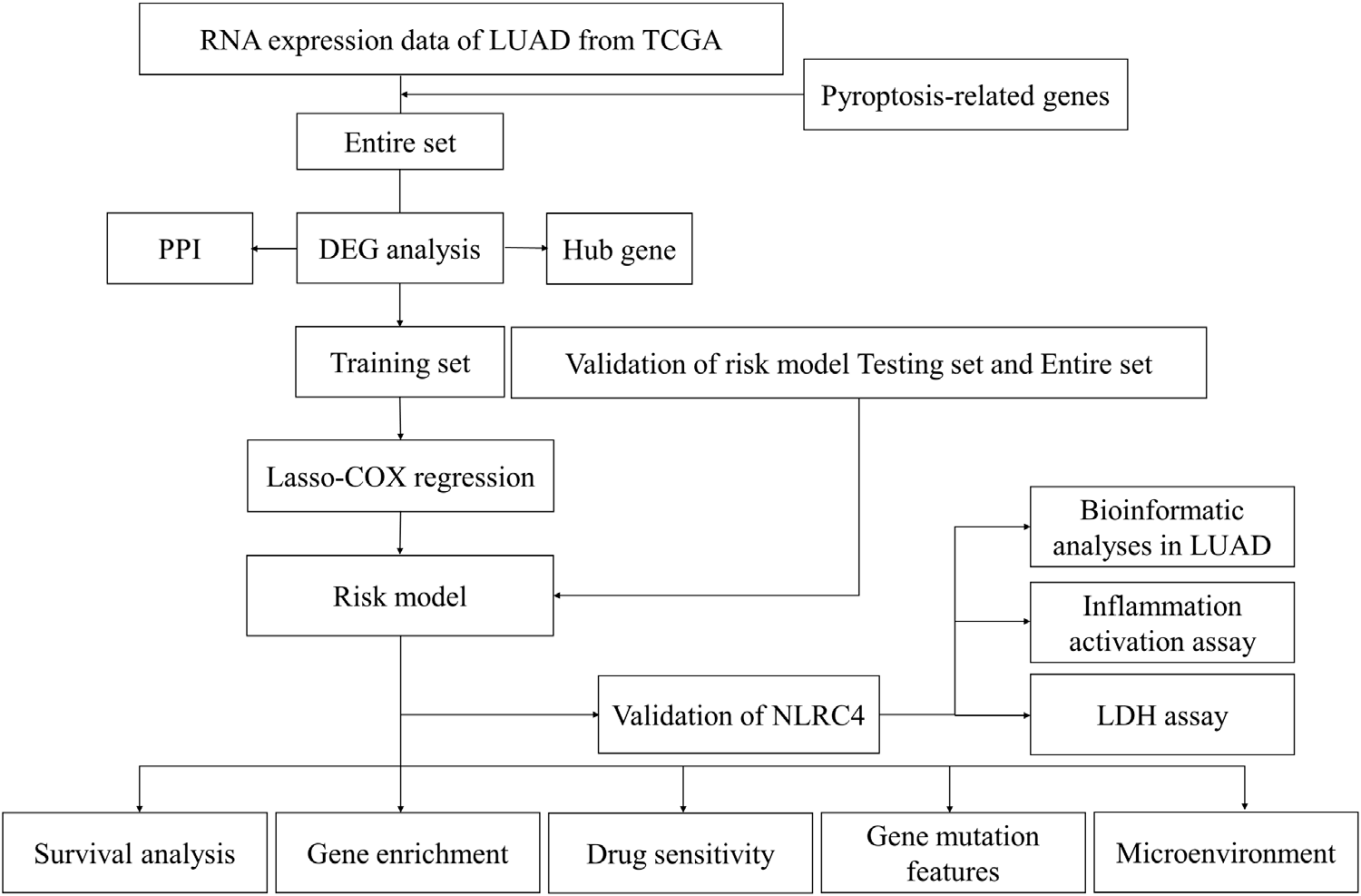
Schematic overview of the workflow in this study.

The LUAD mRNA sequencing data and corresponding clinical data up to 29 April 2022, were obtained from The Cancer Genome Atlas (TCGA) website (https://portal.gdc.cancer.gov). The gene expression profiles were normalized using the “limma” package. 48 PRGs were extracted from the previous study (Wang, et al., 2021). Their information is shown in Table 1. Figure 1 illustrates the complete workflow of the study.

**TABLE 1:** Differences in expression of pyroptosis-related genes between LUAD and normal samples.

Gene symbol	Full name	logFC	p	FDR
AIM2	Absent in melanoma 2	2.956308	7.49E-15	3.00E-14
CARD8	Caspase recruitment domain-containing protein 8	−0.43852	2.48E-11	7.94E-11
CASP1	Caspase 1	−0.7689	2.17E-17	1.16E-16
CASP3	Caspase 3	0.857751	5.28E-26	8.44E-25
CASP4	Caspase 4	0.300571	0.003537	0.005305042
CASP5	Caspase 5	−1.17359	6.90E-20	5.52E-19
CASP6	Caspase 6	1.078872	3.68E-29	8.83E-28
CASP8	Caspase 8	0.563998	1.51E-13	5.56E-13
DDX3X	DEAD-box helicase 3 X-linked	−0.31273	2.68E-06	6.13E-06
GBP1	Guanylate binding protein 1	−0.14397	0.000133	0.000246347
GBP2	Guanylate binding protein 2	0.272685	0.860409	0.860408749
GBP5	Guanylate binding protein 5	0.732585	0.081692	0.095639142
GSDMA	Gasdermin A	1.113508	1.82E-05	3.64E-05
GSDMB	Gasdermin B	1.851111	4.64E-19	3.18E-18
GSDMC	Gasdermin C	2.735457	2.13E-15	9.30E-15
GSDMD	Gasdermin D	0.297322	0.009873	0.013539723
GSDME	Gasdermin E	0.677512	0.013544	0.018058017
GZMA	Granzyme A	0.155768	0.044481	0.054745779
GZMB	Granzyme B	0.117265	0.006247	0.008819273
HMGB1	High mobility group box 1	−0.10315	0.000176	0.000313767
IFI16	interferon γ-inducible protein 16	0.649383	0.000206	0.000352345
IL18	Interleukin 18	−0.2668	0.000113	0.00021771
IL1B	Interleukin 1β	−0.83492	6.83E-13	2.34E-12
IRF1	Interferon regulatory factor 1	−0.50135	1.76E-07	4.45E-07
IRF2	Interferon regulatory factor 2	−0.09168	0.014351	0.018616998
IRF8	Interferon regulatory factor 8	−0.83976	1.29E-15	6.18E-15
MEFV	Mediterranean fever	−1.50059	2.85E-25	3.43E-24
NAIP	neuronal apoptosis inhibitor protein	−0.0802	0.004813	0.007001444
NEK7	NIMA-related kinase 7	−0.75799	5.68E-22	5.45E-21
NLRC3	NOD-like receptor family CARD domain containing 3	−0.03511	0.048797	0.058556187
NLRC4	NOD-like receptor family CARD domain containing 4	−1.89292	1.90E-32	9.11E-31
NLRC5	NOD-like receptor family CARD domain containing 5	0.459784	0.028978	0.036603825
NLRP1	NOD-like receptor (NLR) family pyrin domain-containing 1	−0.31658	9.75E-06	2.03E-05
NLRP12	NOD-like receptor (NLR) family pyrin domain-containing 12	−0.35668	2.91E-08	8.22E-08
NLRP2	NOD-like receptor (NLR) family pyrin domain-containing 2	1.315508	0.652115	0.665990211
NLRP3	NOD-like receptor (NLR) family pyrin domain-containing 3	−0.67298	5.87E-08	1.57E-07
NLRP6	NOD-like receptor (NLR) family pyrin domain-containing 6	0.307339	0.236802	0.258329677
NLRP7	NOD-like receptor (NLR) family pyrin domain-containing 7	2.136525	5.36E-07	1.29E-06
NLRP9	NOD-like receptor (NLR) family pyrin domain-containing 9	0.665345	0.095985	0.109697104
NOD1	Nucleotide-binding oligomerization domain-containing protein 1	−0.25127	3.69E-06	8.05E-06
NOD2	Nucleotide-binding oligomerization domain-containing protein 2	0.051304	0.450026	0.480027341
NR2C2	nuclear receptor subfamily 2, group C, member 2	0.214582	0.593925	0.619747761
P2RX7	P2X purinoceptor 7	−0.8271	1.67E-17	1.00E-16
PKN1	Serine/threonine-protein kinase N1	−0.16294	0.000444	0.000687913
PKN2	Serine/threonine-protein kinase N2	0.230417	0.163305	0.182294012
PYCARD	Apoptosis-associated speck-like protein containing a CARD	−0.2949	0.000231	0.000382215
TNF	Tumor necrosis factor	−0.23493	0.000321	0.000513452
ZBP1	Z-DNA-binding protein 1	1.384278	1.02E-09	3.06E-09

### Identification of differentially expressed PRGs

The differentially expressed genes (DEGs) were extracted based on RNA expression between the normal and tumor samples in the entire cohort. The following criteria were used: |logFC| >0.5 and FDR <0.05. The “pheatmap” package was used to create a heatmap of all these genes. In addition to this, we also performed a preliminary analysis of the correlation among PRGs based on their expression. A protein-protein interaction (PPI) network for DEGs was constructed using the Search Tool for the Retrieval of Interacting Genes (https://string-db.org/). Cytoscape was then used for additional display, and the hub genes were obtained using the Maximal Clique Centrality (MCC) method. The association network of PRGs was used to emphasize the significance.

### Construction and validation of the pyroptosis-related gene prognostic model

In addition, we randomly separated the data into two groups (the training and testing datasets). The clinical data were compared and summarized in Table 2.The associations between each gene and survival status in the TCGA cohort were investigated using Cox regression analysis to establish the predictive value of PRGs. We chose a cutoff *p* value of 0.05 to avoid omissions. The least absolute shrinkage and selection operator (LASSO) method was used for variable selection and shrinkage using the “glmnet” R package in the training dataset to filter the candidate genes and generate the prognostic model. After determining the penalty parameter (*λ*) for the model, the risk scores were computed for each patient based on the expression level of the extracted gene, and the risk score formula was as follows: Risk score = $\sum_{i}^{n}X_{i}\times Y_{i}$ (*X*: coefficients, *Y*: gene expression level).

**TABLE 2:** Comparison of clinical characteristics. (Chi-squared test).

Parameter	Type	Total	Testing	Training	*p* value
Age	<60	137 (27.57%)	68 (27.42%)	69 (27.71%)	0.2666
	≥60	360 (72.43%)	180 (72.58%)	180 (72.29%)	
Gender	FEMALE	268 (53.92%)	144 (58.06%)	124 (49.8%)	0.0787
	MALE	229 (46.08%)	104 (41.94%)	125 (50.2%)	
Stage	Stage I	265 (53.32%)	145 (58.47%)	120 (48.19%)	0.1572
	Stage II	118 (23.74%)	50 (20.16%)	68 (27.31%)	
	Stage III	80 (16.1%)	40 (16.13%)	40 (16.06%)	
	Stage IV	26 (5.23%)	12 (4.84%)	14 (5.62%)	
	unknow	8 (1.61%)	1 (0.4%)	7 (2.81%)	
T	T1	168 (33.8%)	95 (38.31%)	73 (29.32%)	0.1522
	T2	262 (52.72%)	125 (50.4%)	137 (55.02%)	
	T3	45 (9.05%)	18 (7.26%)	27 (10.84%)	
	T4	19 (3.82%)	9 (3.63%)	10 (4.02%)	
	unknow	3 (0.6%)	1 (0.4%)	2 (0.8%)	
N	N0	320 (64.39%)	167 (67.34%)	153 (61.45%)	0.2467
	N1	93 (18.71%)	41 (16.53%)	52 (20.88%)	
	N2	70 (14.08%)	33 (13.31%)	37 (14.86%)	
	N3	2 (0.4%)	2 (0.81%)	0 (0%)	
	unknow	12 (2.41%)	5 (2.02%)	7 (2.81%)	
M	M0	328 (66%)	165 (66.53%)	163 (65.46%)	0.9883
	M1	25 (5.03%)	12 (4.84%)	13 (5.22%)	
	unknow	144 (28.97%)	71 (28.63%)	73 (29.32%)	

### Prognostic value and validation of the risk model

The clinical data (age and stage) of all patients were retrieved and analyzed in conjunction with the risk score. The univariate and multivariate Cox regression models were used to determine the independence of components. The “prcomp” function in the “stats” R package was used to conduct principal component analysis (PCA) based on the gene signature. The sensitivity and specificity of the prognostic model were tested using time-dependent receiver operating characteristic (ROC) analysis. A 1-, 3-, and 5-year ROC curve study was performed using the “survival” “survminer” and “timeROC” R packages. Patients with LUAD were divided into subgroups with low and high risk based on the median risk score. Kaplan-Meier (K-M) analysis was used to evaluate the prognostic value of the risk model, including the overall survival (OS) in all three datasets, and the progression-free survival (PFS) in the whole dataset. This was done to ensure that the risk model was stable. The OS in patients with different stages was also analyzed.

### Functional enrichment analysis of the differentially expressed genes between the low- and high-risk groups

The full cohort of patients with LUAD was separated into two subgroups according to the median risk score. And the clinical features of the two risk-groups were also compared. DEGs between low- and high-risk groups were filtered based on the criteria of |log_2_FC| ≥1 and FDR <0.05. The “clusterProfiler” and “enrichplot” packages were used to conduct Gene Ontology (GO) and Kyoto Encyclopedia of Genes and Genomes (KEGG) enrichment analyses based on these DEGs.

### Genetic and clinicopathological features based on the risk model

TMB represents the number of mutations per million bases in tumor tissues. The tumor tissues with a higher TMB are detected by the immune system more quickly, enhancing the efficacy of immunotherapy (Chan, et al., 2019). The TMB score for each patient with LUAD was calculated using the somatic mutation data of patients obtained from the TCGA database to investigate the association between the expression pattern of PRGs, TMB, and immunity. In addition, the relationship between the TMB and the risk score derived by the stratified model, as well as the impact of these factors on survival were also evaluated. The infiltration score of 16 immune cells and the activity of 13 immune-related pathways were measured using the single-sample gene set enrichment analysis (ssGSEA) function of “gsva” R package. Furthermore, we analyzed the immune cells infiltration with different methods. The Estimation of Stromal and Immunological Cells in Malignant Tumors using the Expression Data (ESTIMATE, https://bioinformatics.mdanderson.org/estimate/index.html) website provided the stromal score, immune score, and ESTIMATE score of samples in the TCGA database, which were applied for further validation.

### Analyses of the sensitivity to anti-cancer therapy based on the model

ICIs are an efficient method for treating various types of cancer. In this study, the expression levels of immune checkpoint molecules, such as cytotoxic T-lymphocyte-associated protein 4 (CTLA-4) and PD-L1, were compared between the two groups to see whether the stratified model could identify patients with LUAD having a favorable response to ICIs. Besides comparing the expression of immune-checkpoint-related genes, we also analyzed the Tumor Immune Dysfunction and Exclusion (TIDE) score to identify ICI-beneficial patients. Following the uploading of the gene expression file as the instruction, the TIDE score was acquired from the website (http://tide.dfci.harvard.edu/). The Cancer Immunome Atlas (TCIA) was applied to conduct comprehensive immunogenomic analyses based on the sequencing data from TCGA. We also used “pRRophetic” R package to evaluate drug sensitivity, which was determined by the concentration that could inhibit 50% of cellular growth (IC50) based on the risk level.

### Bioinformatics validation of NLRC4

We compared the pattern of NLRC4 expression in pan-cancer. Also, we separated the complete dataset into low- and high-expression groups according to the mRNA level of NLRC4 in LUAD. GO enrichment and KEGG analyses were performed on the basis of the DEGs obtained from the two groups. The correlations with the LUAD microenvironment, including TMB, immune cell infiltrations, and immune checkpoints were also evaluated. Additionally, clinical factors, such as survival and medication sensitivity were applied to investigate its value in clinical. The methods used here were mentioned above. In addition, we acquired the immunophenoscore (IPS) for each LUAD from the Cancer Immunome Database (https://tcia.at/home). It served as a predictor of anti-CTLA-4 and anti-PD-1 treatment response (Charoentong, et al., 2017). And we compared the differences in IPS between the low- and high-expression groups.

### Cell culture and inflammasome activation assay

Human embryonic kidney 293 cells (HEK-293T) were cultured on Dulbecco’s modified Eagle’s medium (DMEM, Gibco, US) containing 10% fetal bovine serum (Gibco, US) and antibiotics (penicillin and streptomycin). H1299 cells were cultured in an incubator at 37°C in the presence of 5% CO_2_ with Roswell Park Memorial Institute1640 (RPMI-1640, Gibco, US) containing 10% fetal bovine serum and antibiotics. HEK-293T or H1299 cells were plated into six-well tissue culture plates overnight. HEK-293T cells were transfected with Polyethylenimine and Human influenza hemagglutinin (HA) tagged full-length NLRC4, pro-caspase 1, apoptosis-related specific protein (ASC), and pro-interleukin (IL)-1β to imitate the activation of the inflammasome, while H1299 cells were transfected with HA-tagged full-length NLRC4 for the lactate dehydrogenase (LDH) assay. The cells were collected for further study after 48 h incubation.

### Lactate dehydrogenase (LDH) assay

LDH is a stable cytoplasmic enzyme present in every cell. When the plasma membrane is compromised, LDH is rapidly released into the culture supernatant. LDH leakage was evaluated using a colorimetric LDH test kit (Promega, US) following the manufacturer’s protocols. The absorbance value of each group was compared with the absorbance value of the control group.

### Quantitative polymerase chain reaction and Western blotting

RNA was extracted using TRIzol (ThermoFisher, US) following the manufacturer’s protocols. The isolated RNA was reverse-transcribed into cDNA using a first-strand cDNA synthesis kit (ABclonal, CN). Quantitative PCR was performed in triplicate using the MonAmp SYBR Green qPCR Mix kit (Monad, CN), and was performed on an RT-PCR instrument (Bio-RAD, US). Glyceraldehyde 3-phosphate dehydrogenase (GAPDH) was used as a reference.

The primer sequences for qPCR were as follows:

RT-PCR GAPDH Forward 5′-TGA​CTT​CAA​CAG​CGA​CAC​CCA-3′

RT-PCR GAPDH Reverse 5′-CACCCTGTTGCTGT AGCCAAA-3′

RT-PCR NLRC4 Forward: 5′-GTG​TTC​TCC​CAC​AAG​TTT​GA-3′

RT-PCR NLRC4 Reverse: 5′-AGT​AAC​CAT​TCC​CCT​TGG​TC-3′

RT-PCR caspase-1 Forward: 5′-CAG​ACA​AGG​GTG​CTG​AAC​AA -3′

RT-PCR caspase-1 Reverse: 5′ -TCG​GAA​TAA​CGG​AGT​CAA​TCA-3′

RT-PCR IL-1β Forward 5′-ATG​GCA​GAA​GTA​CCT​GAG​CTC-3′

RT-PCR IL-1β Reverse 5′-TTA​GGA​AGA​CAC​AAA​TTG​CAT​G-3′

The cells were collected and lysed with 2% sodium dodecyl sulfate (SDS) in the presence of complete protease inhibitor mixture. The loading volume was mainly adjusted by the expression of tubulin. After adjusting by 1 × loading buffer, equal amounts of protein were loaded onto SDS-PAGE gels and transferred to NC membranes. The membranes were blocked for 1 h at RT with 5% milk and subsequently incubated at 4°C overnight with the primary antibody. After washing the membrane, incubated with secondary antibody for 1 h at RT. Then, washed the membrane again before chemiluminescence analysis. The following antibodies were used: HA (CST, United States, cat#3724S), caspase-1 (CST, United States, cat#3866T), IL-1β (CST, United States, cat#12242S), and tubulin (proteintech, CN, cat#I0004491). The secondary anti-mouse (proteintech, CN, cat# SA00001-1) and anti-rabbit immunoglobulins (proteintech, CN, cat# SA00001-4) were used.

### Statistical analysis

All statistical analyses in this study were conducted on R software (version 4.1.2). The statistical tests were all two sided. A *p* value < 0.05 indicated a statistically significant difference. The statistical significance of two groups was evaluated using the Student *t* test and Wilcoxon test. For correlation analysis was conducted by Spearman and Pearson cor test. Chi-squared test was used to caculate composition difference.

## Results

### Identification of differentially expressed genes between normal and tumor tissues

**FIGURE 2:**
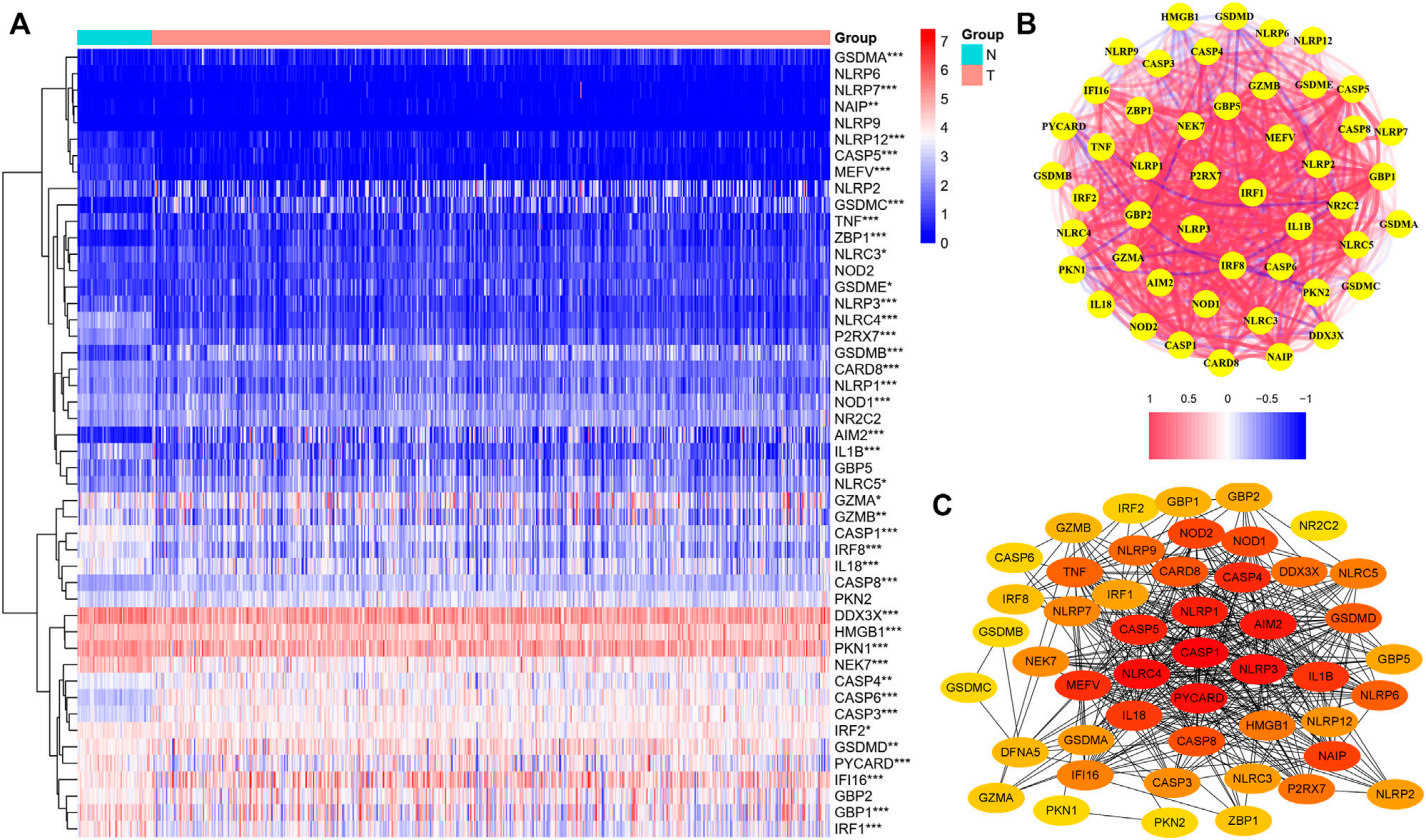
Expression of the 48 pyroptosis-related genes and the interactions among them. **(A)** Heatmap of the pyroptosis-related genes between the normal (N, brilliant blue) and tumor tissues (T, red). (wilcox.test, *p* values were as follows: ^**^ *p* < 0.01; ^***^ *p* < 0.001.) **(B)** PPI network showing the interactions of the differentially expressed pyroptosis-related genes (red line: positive correlation; blue line: negative correlation). **(C)** Correlation network of all the pyroptosis-related genes. The intensity of the colors reflected the strength of the relevance.

We found that 21 of the 48 PRGs were differentially expressed (logFC >0.5, FDR <0.05) in the TCGA dataset consisting of 59 normal and 535 tumor tissues; 10 genes were downregulated (*IL-1β*, *NLRC4*, *CASP5*, *IRF1*, *CASP1*, *NLRP3*, *NEK7*, *P2RX7*, *MEFV*, and *IRF1*), while the remaining were upregulated (*CASP8*, *GSDME*, *CASP3*, *CASP6*, *GSDMA*, *GSDMB*, *NLRP7*, *GSDMC*, *ZBP1*, *IFI16*, and *AIM2*). The RNA levels of these genes are depicted as heatmaps in Figure 2A. The correlation network containing all PRGs is shown in Figure 2B (the red line represents positive correlations, while the blue line represents negative correlations), and most of these genes displayed a positive relationship. We conducted a PPI analysis for these genes to further investigate the interactions of these PRGs. Among these, *CASP1*, *NLRC4*, *NLRP1*, *CASP5*, *NLRP3*, *CASP8*, and *AIM2* were the hub genes, which had the maximum interactions with other genes. The results are presented in Figure 2C.

### Development of a prognostic gene model in the TCGA training cohort and validation

**FIGURE 3:**
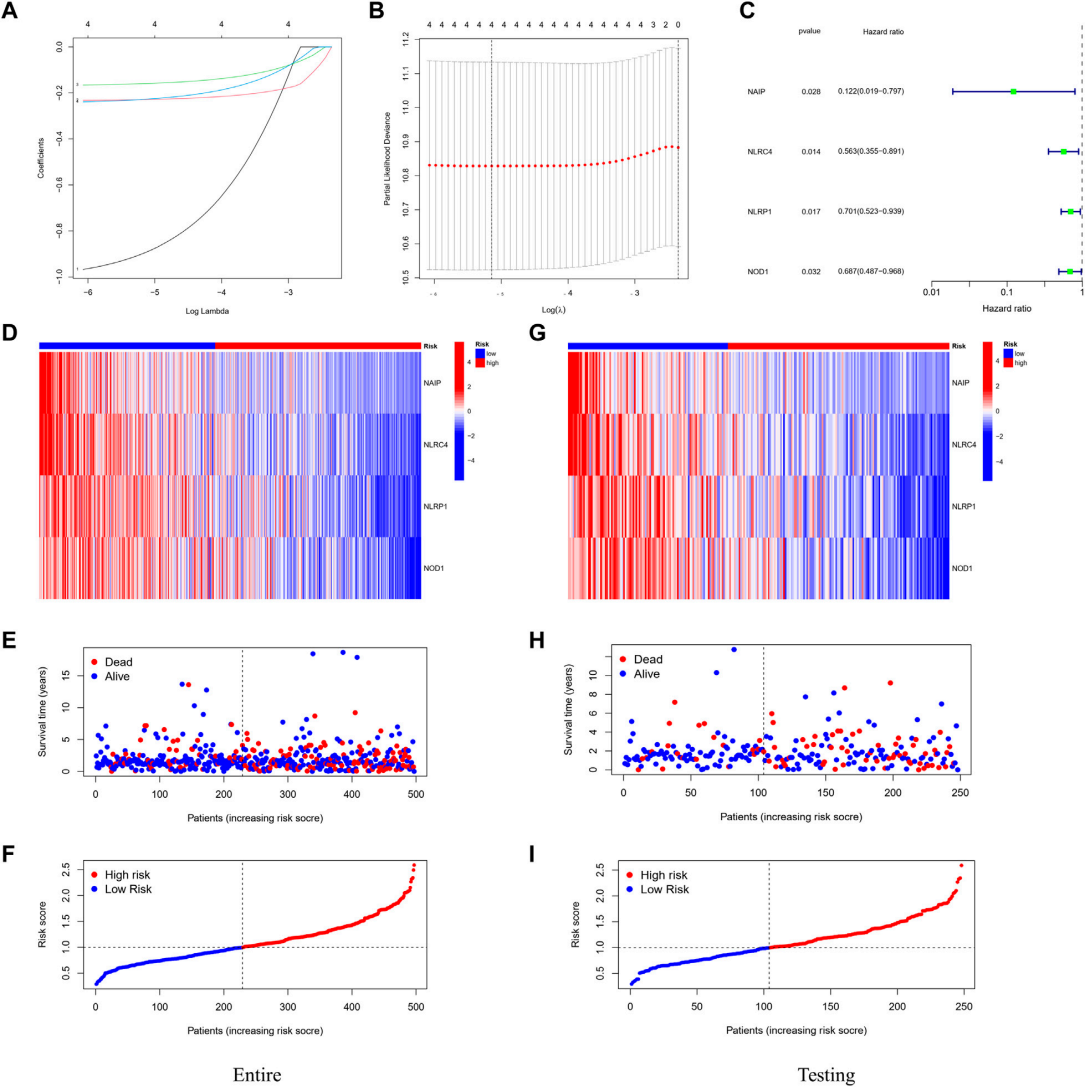
Construction of the prognostic stratification model. **(A)** LASSO coefficient profiles. **(B)** Four candidate genes obtained by LASSO regression. **(C)** Four optimal genes obtained by multivariable Cox regression analysis. **(D)** Expression pattern of the four optimal genes between tumor and normal specimens in the entire dataset. **(E)** Survival status for each patient (low-risk population: on the left side of the dotted line; high-risk population: on the right side of the dotted line). **(F)** Distribution of patients based on the risk score. **(G–I)** Expression pattern of the four optimal genes; survival status and distribution of patients based on the risk score between tumor and normal specimens in the testing dataset.

A total of 497 samples with complete survival information were collected for further analysis. Initially, the genes associated with survival were evaluated using univariate Cox regression analysis on the training dataset. We retained the four genes *NLRC4*, NOD-like receptor (NLR) family pyrin domain-containing 1 (*NLRP1*), Nucleotide-binding oligomerization domain-containing protein 1 (*NOD1*), and NLR family apoptosis inhibitory proteins (*NAIP*) for risk model development based on the optimal value from LASSO Cox regression analysis (Figures 3A–C). The risk score formula was: Risk score = (−1.01608731367098 × NAIP exp) + (–0.235231123651649 × NLRC4 exp) + (–0.169828624865761 × NLRP1 exp) + (–0.246566494613787 × NOD1 exp). In addition, patients in various datasets were divided into low- and high-risk subgroups based on the median risk score. And the clinical parameters comparisons in the entire cohort were shown in Table 3. All these genes were relatively suppressed in the population at high-risk. Patients in the high-risk group (on the left side of the dashed line) died more often and lived shorter times than those in the low-risk group (on the right side of the dashed line). This showed that LUAD with a high score might have a worse outcome. Additionally, Figures 3D–F shows the whole dataset, and Figures 3G–I shows the testing dataset.

**TABLE 3:** comparison of clinical features between two-risk groups in entire set. (Chi-squared test).

Parameter	Type	Total	High	Low	*p* value
Age	<60	133 (27.2%)	77 (29.39%)	56 (24.67%)	0.2856
	≥60	356 (72.8%)	185 (70.61%)	171 (75.33%)	
Gender	FEMALE	265 (54.19%)	133 (50.76%)	132 (58.15%)	0.1226
	MALE	224 (45.81%)	129 (49.24%)	95 (41.85%)	
Stage	Stage I	265 (54.19%)	125 (47.71%)	140 (61.67%)	0.0115
	Stage II	118 (24.13%)	68 (25.95%)	50 (22.03%)	
	Stage III	80 (16.36%)	51 (19.47%)	29 (12.78%)	
	Stage IV	26 (5.32%)	18 (6.87%)	8 (3.52%)	
T	T1	167 (34.15%)	73 (27.86%)	94 (41.41%)	0.0154
	T2	257 (52.56%)	151 (57.63%)	106 (46.7%)	
	T3	44 (9%)	27 (10.31%)	17 (7.49%)	
	T4	18 (3.68%)	10 (3.82%)	8 (3.52%)	
	unknow	3 (0.61%)	1 (0.38%)	2 (0.88%)	
N	N0	315 (64.42%)	154 (58.78%)	161 (70.93%)	0.0046
	N1	91 (18.61%)	58 (22.14%)	33 (14.54%)	
	N2	70 (14.31%)	46 (17.56%)	24 (10.57%)	
	N3	2 (0.41%)	0 (0%)	2 (0.88%)	
	unknow	11 (2.25%)	4 (1.53%)	7 (3.08%)	
M	M0	322 (65.85%)	184 (70.23%)	138 (60.79%)	0.2148
	M1	25 (5.11%)	18 (6.87%)	7 (3.08%)	
	unknow	142 (29.04%)	60 (22.9%)	82 (36.12%)	

### Independent prognostic value of the risk model and its clinical application

**FIGURE 4:**
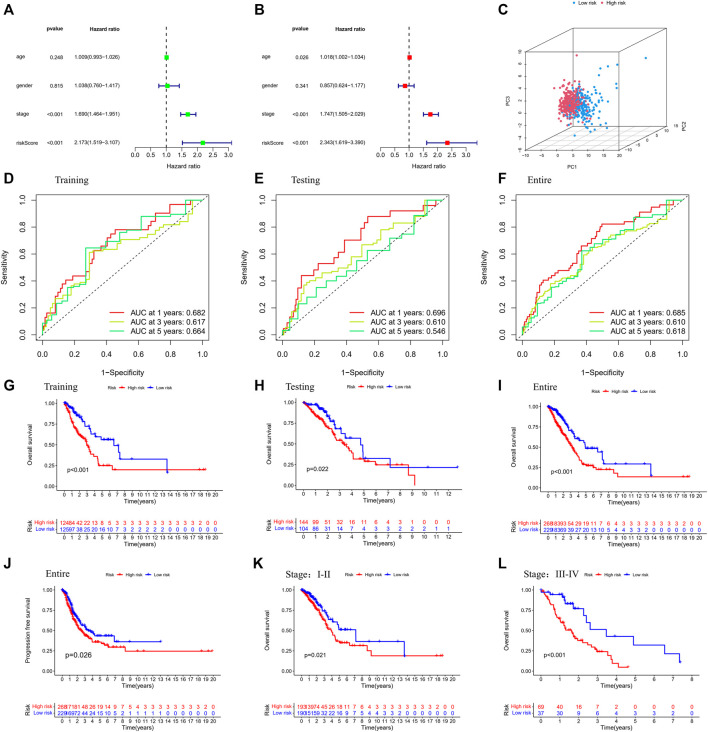
Validation of the prognostic stratification model. **(A)** Univariate analysis for the entire dataset. **(B)** Multivariate analysis for the entire dataset. **(C)** PCA plot for LUAD in the entire dataset. **(D–F)** Time-dependent ROC curves for LUAD in the training, testing, and entire datasets. **(G–I)** K-M survival analysis of different risk groups in the training, testing, and entire datasets. **(J)** PFS of the entire dataset. **(K,L)** K-M survival analysis of patients with different stages in low- and high-risk groups. (Chi-squared test).

The univariate and multivariable Cox regression analyses were conducted to determine whether the risk score generated by the gene signature model could be employed as an independent prognostic factor. Both the stage and the risk score were found to be independent predictors of poor survival in the TCGA cohorts *via* the univariate Cox regression analysis (*p* < 0.001, Figure 4A,B). We also performed PCA based on tumor and normal specimens and found that the 48 PRGs completely distinguished LUAD samples (Figure 4C). The area under the ROC curve was 0.685 for 1-year survival, 0.610 for 3-year survival, and 0.618 for 5-year survival (Figure 4D). They were further confirmed in the testing and entire datasets, indicating that the pyroptosis-associated risk score had a strong and dependable capacity to predict the prognosis for patients with LUAD. All three datasets showed poorer OS in the high-risk group (*p* < 0.05), as well as the PFS in the entire dataset (*p* = 0.026). These results demonstrated that patients with a high-risk score had a worse prognosis (Figure 4G–J). We further examined the risk model by comparing the OS in patients with different stages. We discovered that patients in the high-risk group had a worse prognosis for both early and advanced stages of lung cancer (Figure 4K,L: *p* = 0.021 in stages I and II, *p* < 0.001 in stages III and IV).

### Distinct biological processes, TME, and treatment decision characteristics in LUAD were based on the risk model

**FIGURE 5:**
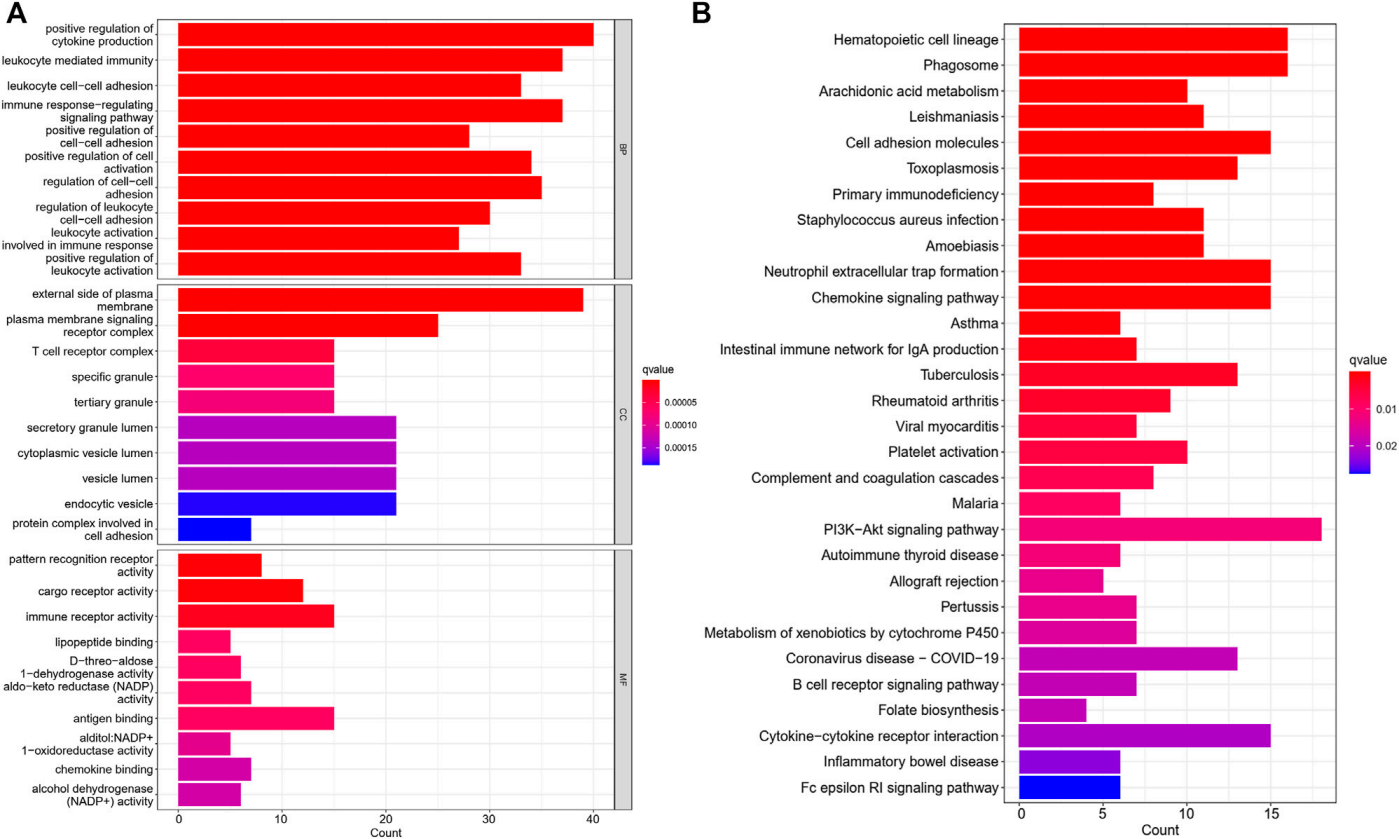
The GO and KEGG analyses between the two risk groups **(A)** Bar graph for GO enrichment. **(B)** Bar graph for KEGG pathways (the longer bar means the enrichment of more genes, and the increasing intensity of red color means more obvious differences; q-value: the adjusted *p* value).

**FIGURE 6:**
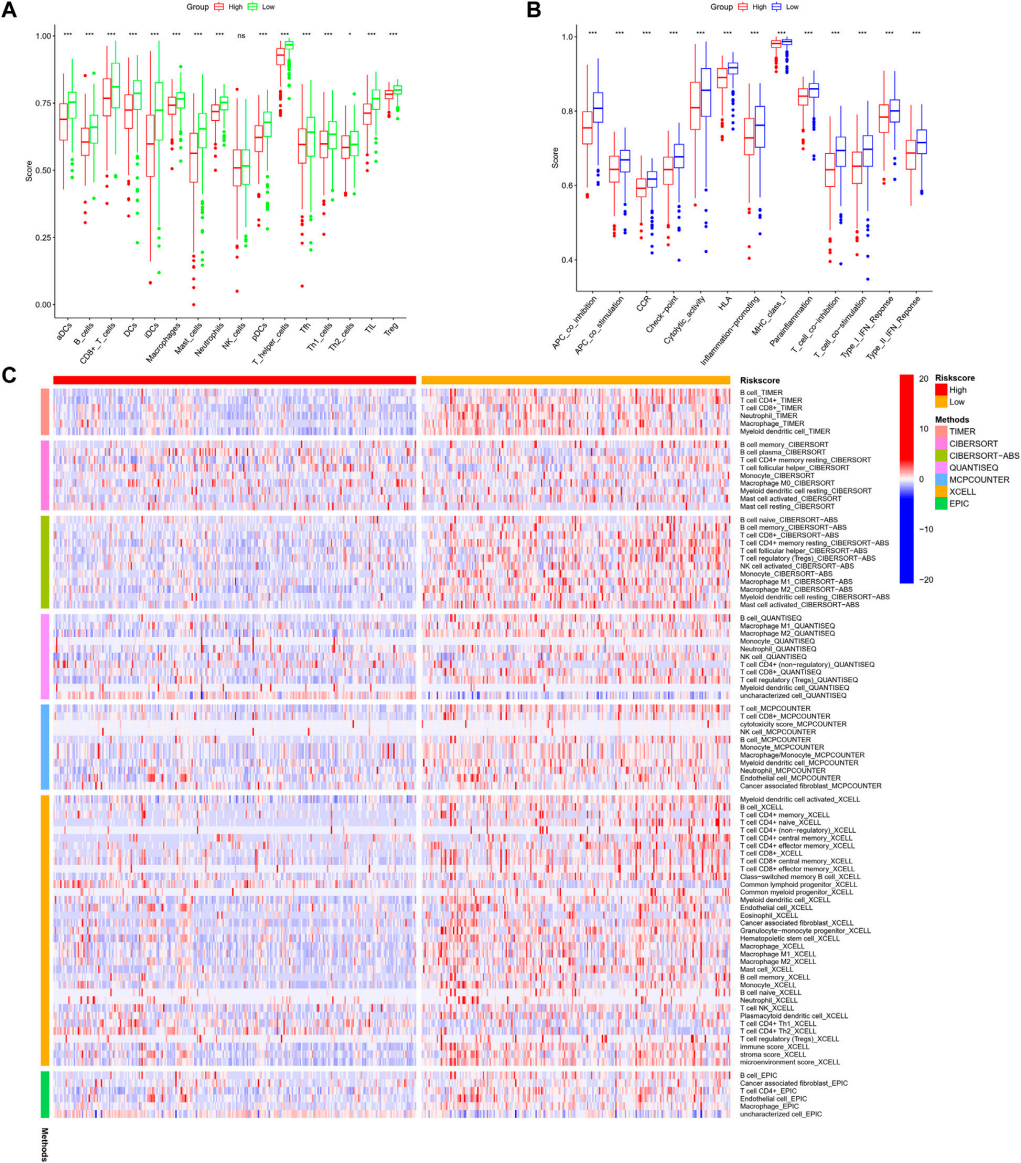
**(A,B)** Comparison of the enrichment scores of 16 types of immune cells and 13 immune-related pathways based on the ssGSEA scores between low- (green box) and high-risk (red box) groups in the TCGA cohort. **(C)** Comparison of the high- and low-risk groups using different methods. (wilcox.test, *p* values were as follows: ^*^ *p* < 0.05; ^**^ *p* < 0.01; ^***^ *p* < 0.001).

Further, we identified DEGs to examine the differences in gene functions and pathways between the subgroups divided by the risk model. Between these, 508 DEGs were identified in the total TCGA cohort. The GO enrichment analysis and KEGG pathway analyses were performed. The GO enrichment of DEGs was primarily associated with the immune system, including cytokine generation, immune response regulation, chemokine binding, and inflammatory cell chemotaxis (Figure 5A). Regarding the KEGG pathway, we discovered that it corresponded with the GO analysis, which included the chemokine signaling pathway, the B cell receptor signaling pathway, the cytokine-cytokine receptor interaction, and so on (Figure 5B). It was obvious that this pyroptosis-related model might be related to immunity, which could help us differentiate LUAD with varying immunological status. The ssGSEA was used to evaluate the enrichment scores of 16 types of immune cells and the activity of 13 immune-related pathways. The low-risk subgroup showed higher proportions of CD8^+^ T cells, neutrophils, natural killer (NK) cells, T helper (Th) cells (Th1 and Th2), tumor-infiltrating lymphocytes, and regulatory T (Treg) cells compared with the high-risk subgroup (Figure 6A). In the entire TCGA cohort, the activity of all 13 immunological pathways was lower in the high-risk group (Figure 6B). The immune cell infiltration was further investigated using various techniques. Consistent with previous findings, many immune-infiltrating cell subpopulations, including effector memory B cells, CD8^+^ T cells, CD4^+^ T cells, and NK cells, were significantly enriched in the low-risk group (Figure 6C).

**FIGURE 7:**
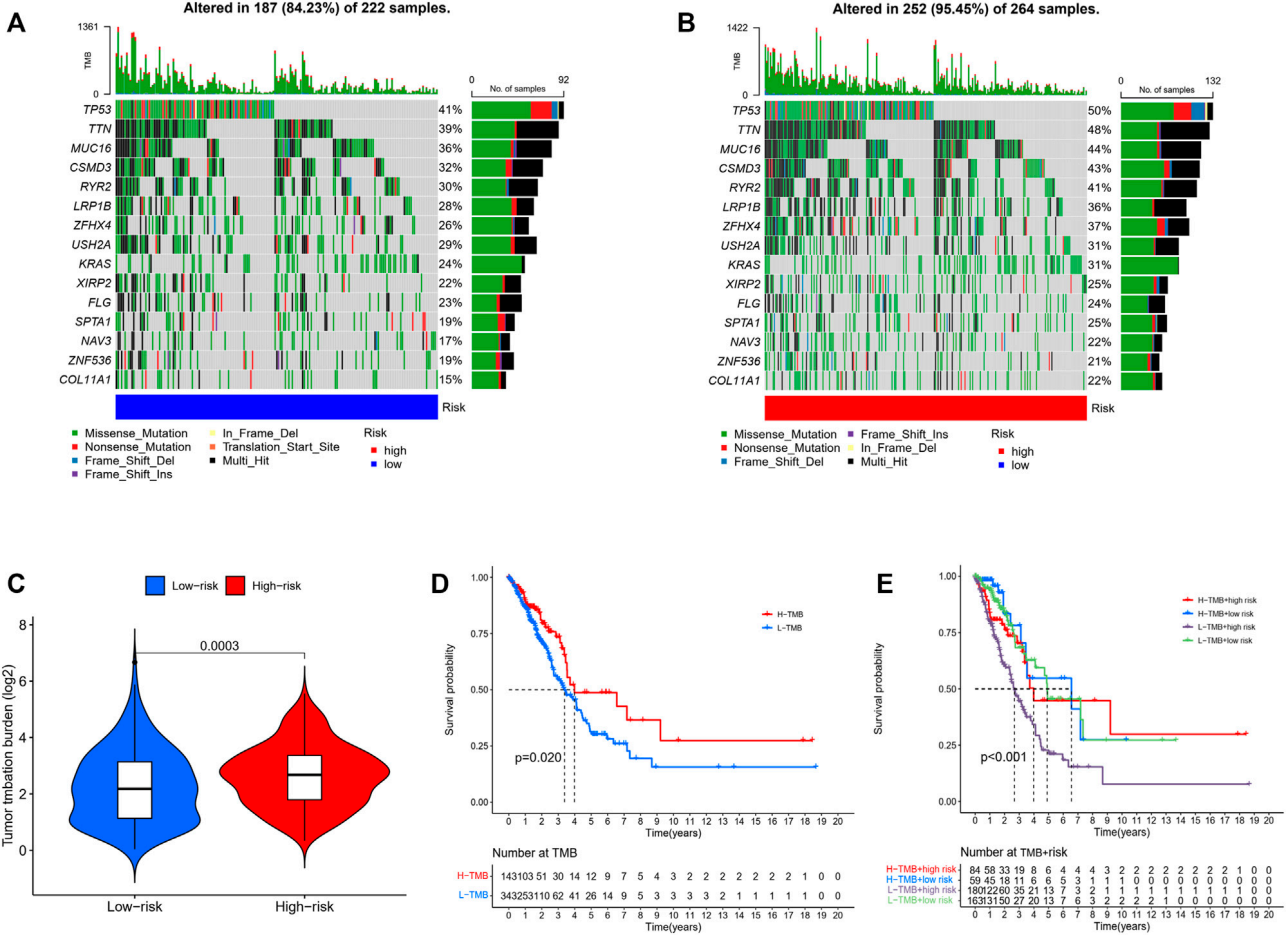
**(A,B)** Mutations in common genes between low- and high-risk groups. **(C)** Comparison of the tumor mutation burden between low- and high-risk groups. (Wilcoxon test) **(D)** Comparison of the survival probability with different levels of tumor mutation burden. **(E)** The survival probability with different levels of tumor mutation burden and risk score. (Chi-squared test).

We analyzed the correlations between the risk model and TMB. To highlight the importance of risk score. We compared the most prevalent mutation genes between the two risk groups by collecting the LUAD mutation data from TCGA. We found that the high-risk group had a significantly greater mutational rate (*p* = 0.0003, Figure 7A–C). The top list included Tumor protein P53 (TP53) and Kirsten rat sarcoma virus (KRAS). Previous studies found that the TP53 gene was a suppressor gene, its mutation had a significant impact on cancer risk, while KRAS mutation correlated with a low response rate to gefitinib in LUAD (Greathouse, et al., 2018; Reck, et al., 2021). The mutations of these oncogenes based on the risk model might indicate some potential relations in the drug resistance of LUAD. Next, the TMB survival probability was explored. Patients with a low TMB and a high-risk level had the worst outcome (Figure 7D,E).

**FIGURE 8:**
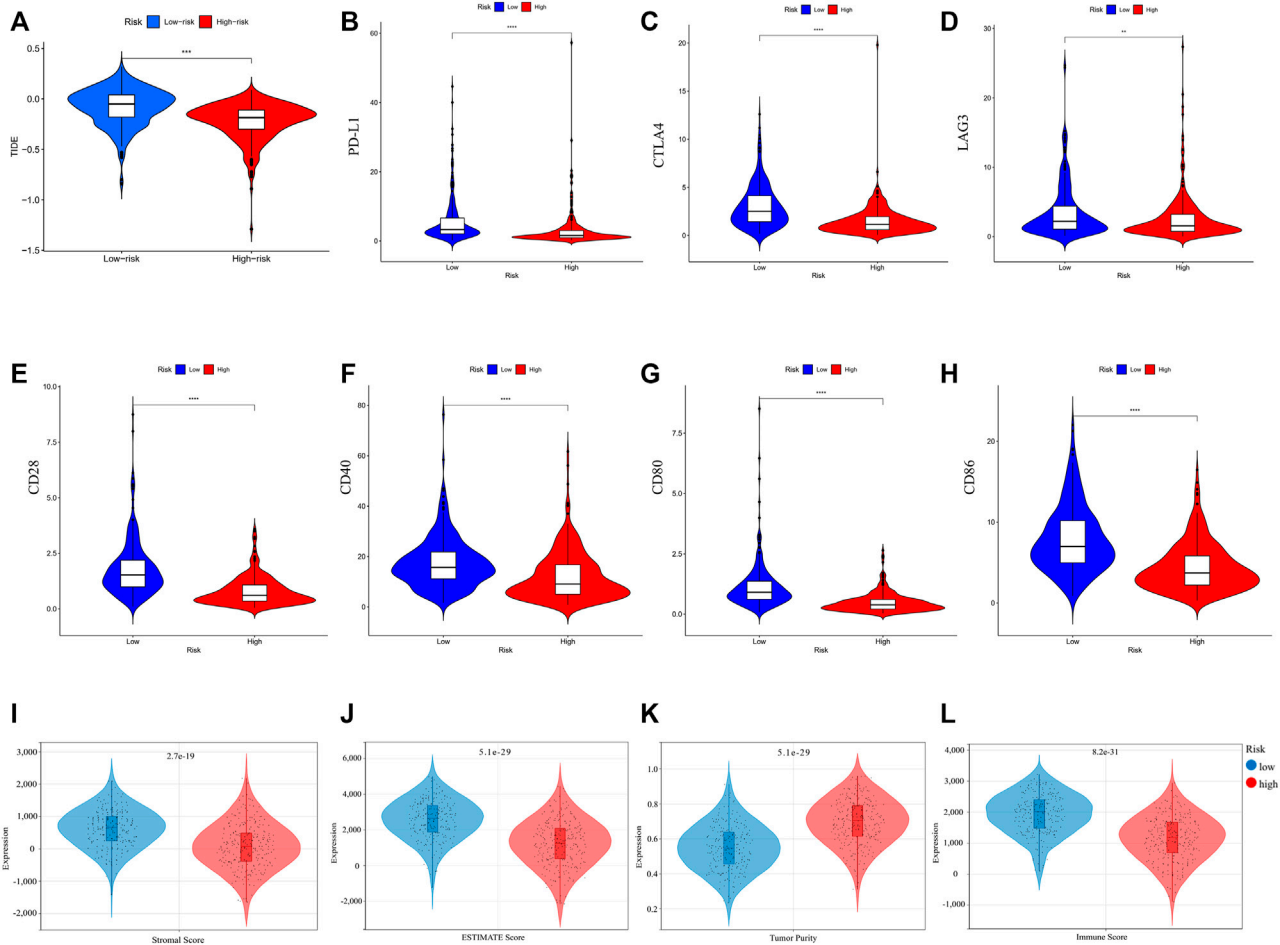
**(A)** TIDE score of different risk groups. **(B–H)** Comparison of immune checkpoint expression between different risk groups. **(I–L)** Tumor immune microenvironment of LUAD comparisons between high-risk and low-risk groups. **(I)** stromal score; **(J)** ESTIMATE score; **(K)** tumor purity; and **(L)** immune score. (wilcox.test, *p* values were as follows: ^*^ *p* < 0.05; ^**^ *p* < 0.01; ^***^ *p* < 0.001).

The correlation between risk score and immune checkpoint expression was further estimated based on the results of GO and KEGG enrichment. As shown in Figure 8A–H, low-risk LUAD exhibited higher expression of various molecular markers (PD-L1, CTLA4, Lymphocyte-activation gene 3: LAG3, CD27, CD80), indicating a superior immunotherapeutic response. Another newly identified predictor TIDE is frequently employed and strongly advised for evaluating the immune response and immune evasion (Jiang, et al., 2018). However, our study revealed that the TIDE expression dramatically increased in the low-risk group, indicating an immune escape phenotype in low-risk group, which is contradictory to immune checkpoint analysis and will be discussed in detail below. In addition, we examined the degree of immune cell infiltration (immune score) and stromal cell infiltration (stromal score) across three unique patterns. The high-risk patients had the lowest immune score compared with the low-risk patients. Also, they had a lower stromal score, indicating that high-risk LUAD had fewer nontumor components, such as immune cells and stromal cells, which might correspond with a poorer response to immunotherapy (Figure 8I–L). The aforementioned results demonstrated that the difference in tumor pyroptosis patterns might play a crucial role in mediating the clinical response to ICIs treatment through the impact on TMB, immune cell infiltration, immunogenicity, and checkpoint expression, providing insights into the crucial role of pyroptosis in the regulation of the immune microenvironment of LUAD.

**FIGURE 9:**
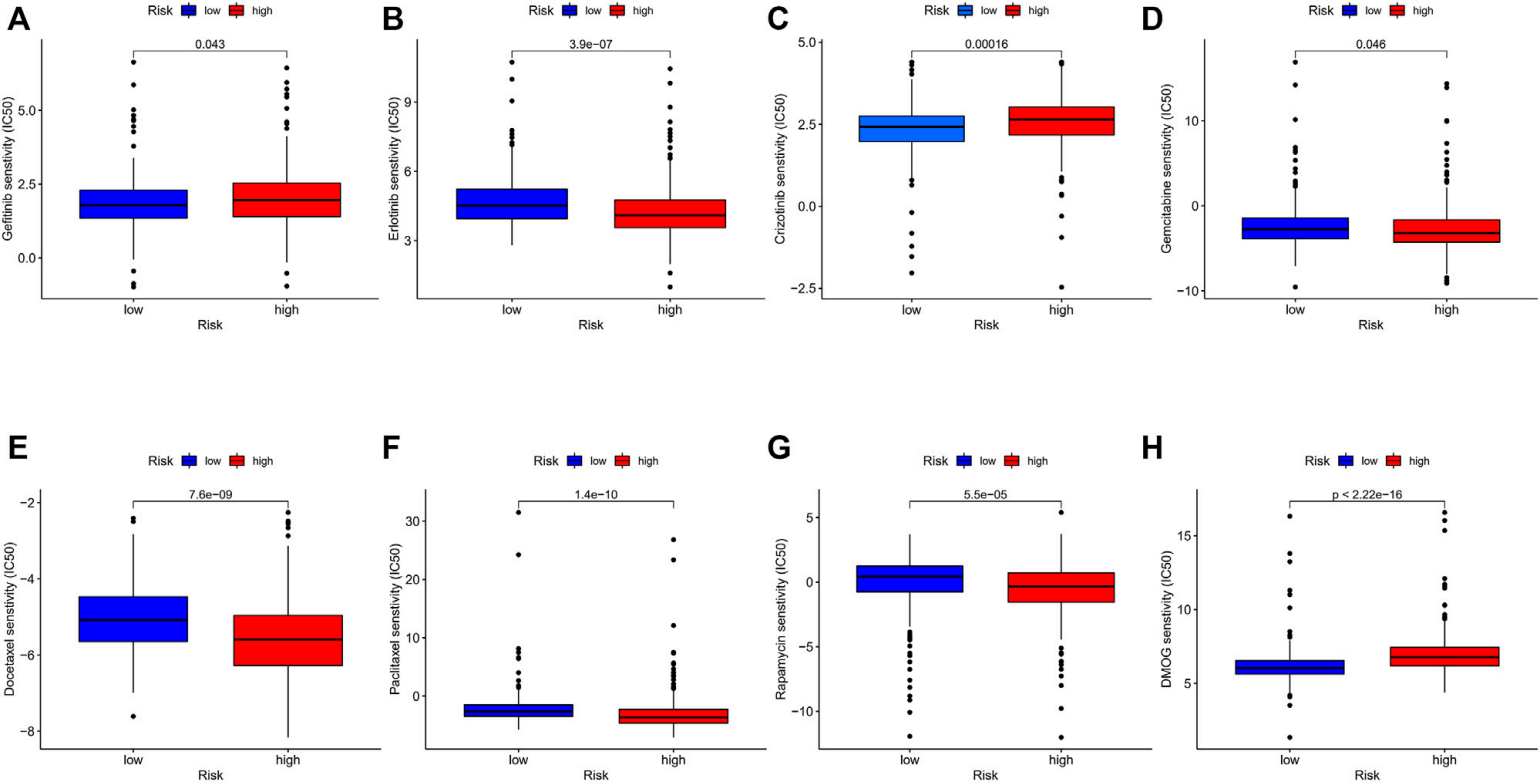
**(A–H)** Comparison of IC50 between different risk groups. (wilcox.test, *p* values were as follows: *p < 0.05; **p < 0.01; ***p < 0.001).

We investigated common anti-tumor drugs in LUAD to confirm whether the PRGs-related risk model could provide treatment suggestions. We discovered that the high-risk group was more sensitive to erlotinib, gemcitabine, docetaxel, paclitaxel, and rapamycin than the low-risk group, while patients with low-risk were more sensitive to gefitinib and crizotinib (Figure 9).

### NLRC4 induced caspase-1-dependent pyroptosis and could arrest the progression of LUAD

**FIGURE 10:**
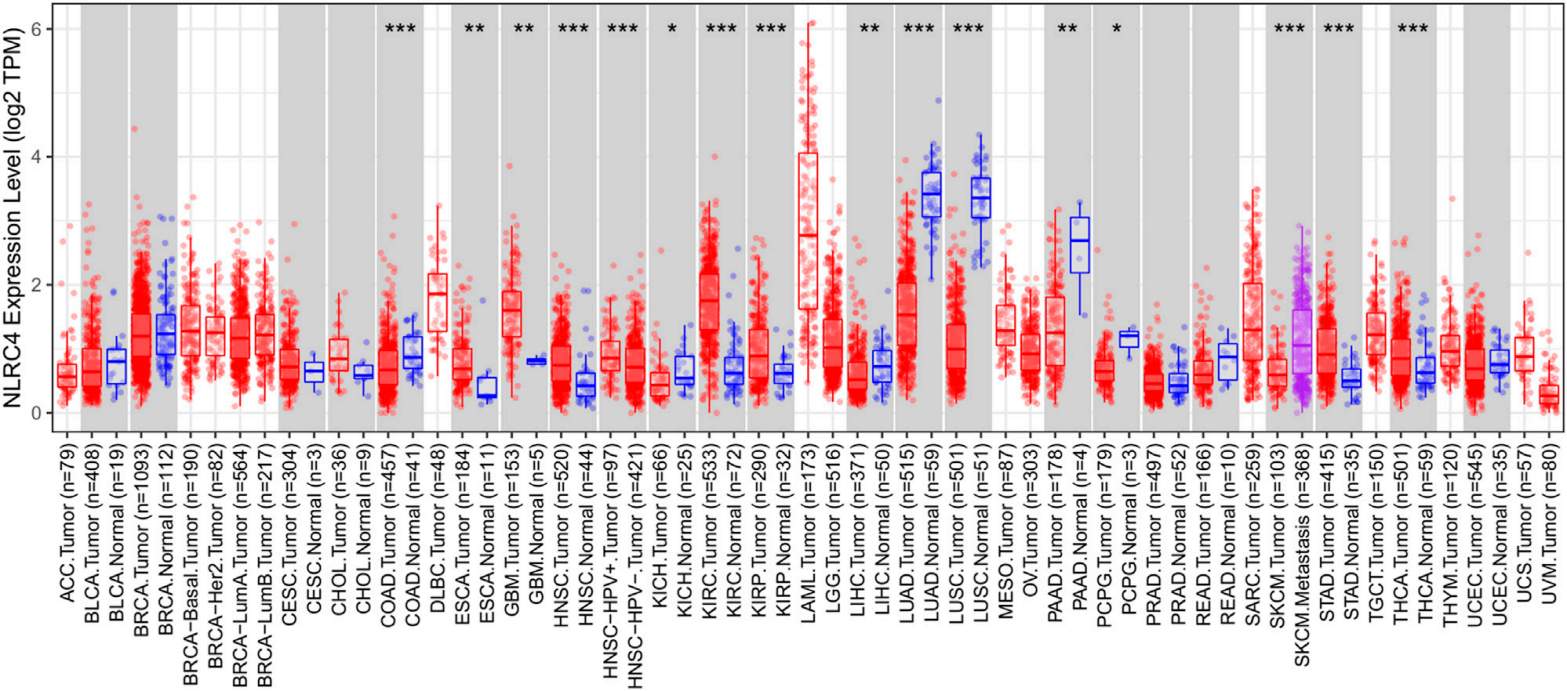
Expression of NLRC4 in pan-cancer compared with normal samples. Expression of NLRC4 was significantly lower in LUAD samples than in normal samples.

**FIGURE 11:**
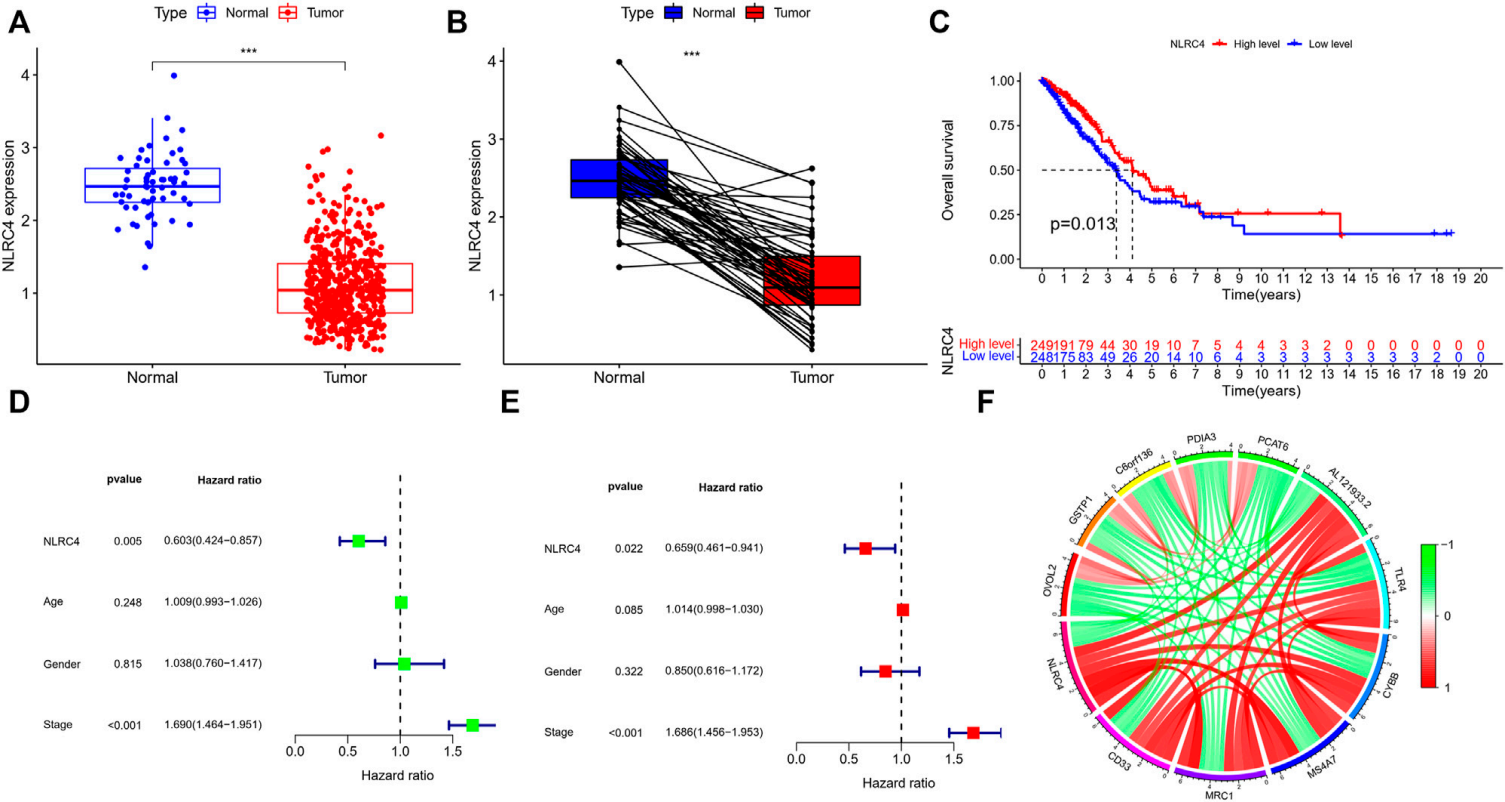
**(A,B)** Expression of NLRC4 in TCGA cohort compared with normal samples (wilcox test, ^***^ *p* < 0.001). **(C)** K-M survival analyses based on the expression of NLRC4 in the LUAD-TCGA cohort. (Chi-squared test) **(D,E)** Univariate and multivariate analyses of different parameters related to survival. **(F)** The correlation of NLRC4 with other genes (red line: positive correlation; green line: negative correlation).

**FIGURE 12:**
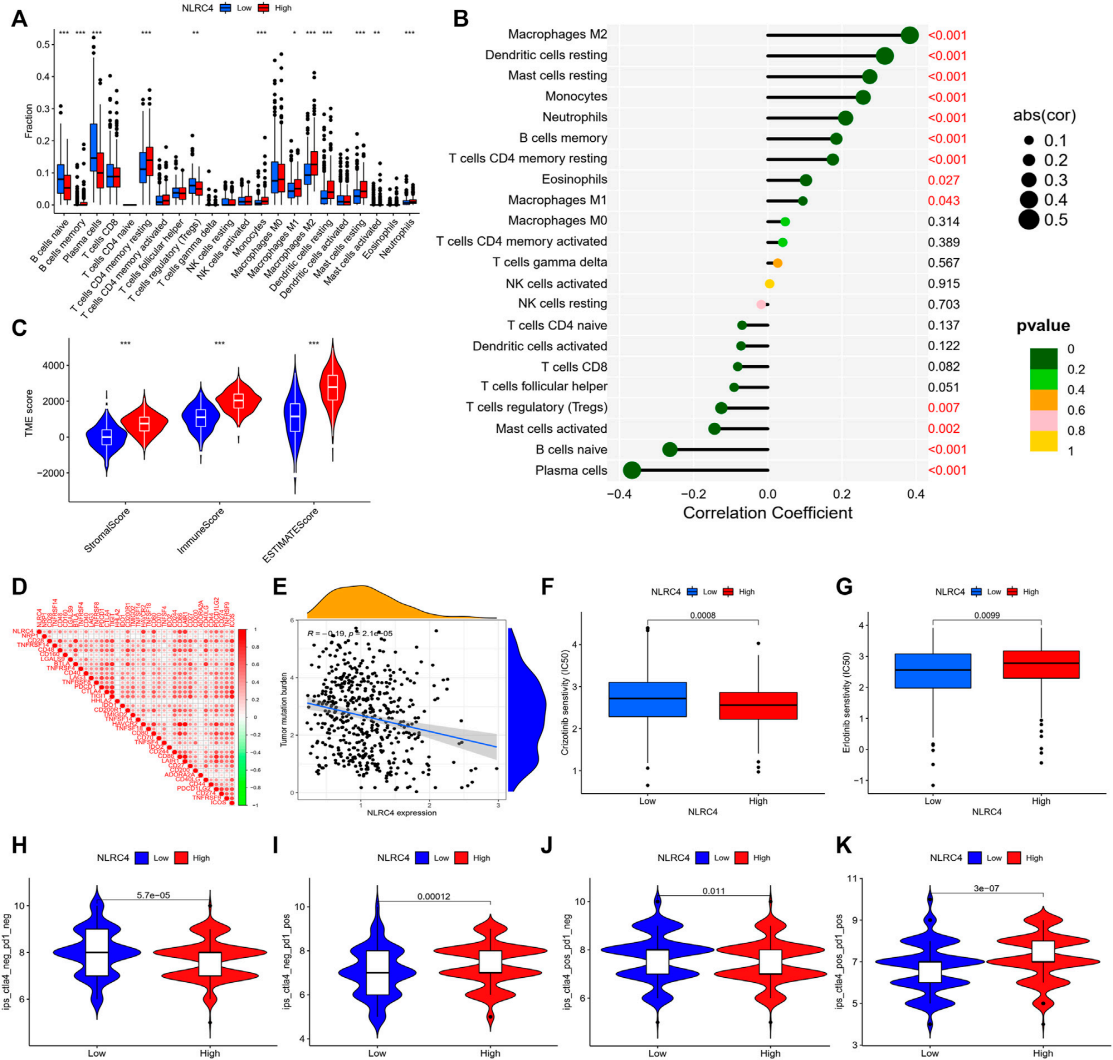
**(A)** Infiltrating levels of 22 immune cell types between high- and low-expression groups. **(B)** Correlation coefficient analyses of NLRC4 and different immune cells. **(C)** Tumor microenvironment between high- and low-NLRC4-expression groups (wilcox test, ^***^ *p* < 0.001). **(D)** Correlation of NLRC4 and other immune checkpoints (Pearson cor test). **(E)** Correlation coefficient analyses of NLRC4 and TMB (Spearman cor test). **(F,G)** Drug sensitivity for LUAD with different NLRC4 expression levels. **(H–K)** TCIA analyses of LUAD with different NLRC4 expression levels. (wilcox test, *p* values were as follows: ^*^ *p* < 0.05; ^**^ *p* < 0.01; ^***^ *p* < 0.001).

We extracted NLRC4 for further investigation to find the potential mechanisms associated with pyroptosis in LUAD. We compared the expression level of NLRC4 in pan-cancer and found that it was much lower in LUAD tissues than in normal tissues (Figure 10). It was validated in the TCGA cohort, where its expression was inversely linked with the outcome (Figure 11A–E). It also had a positive relationship with Toll-like receptor 4 (TLR4), which might promote the synthesis or release of pro- and anti-inflammatory cytokines and chemokines *via* the activation of transcription factors such as NF-κB, as well as the activation of adaptive immunity (Figure 11F) (Pinto, et al., 2011). Furthermore, we investigated the relationship between NLRC4 and immunity. NLRC4 expression negatively correlated with the proportions of Tregs, naive B cells, and plasma cells, and positively correlated with the proportions of dendritic cells, macrophages, and so on (Figure 12A,B). Regarding the tumor tissues, the higher the level of NLRC4, the higher the immune score was (Figure 12C). The expression of NLRC4 was also found to have some correlations with immune checkpoints, especially a positive correlation with CD40 and CD28, which participated in T cell activation. However, the correlation between NLRC4 and TMB was negative (*p* < 0.01) (Figure 12D,E). The drug sensitivity analyses revealed that LUAD with a higher level of NLRC4 was more sensitive to crizotinib (Figure 12F,G), which needs clinical data for validation. The immunotherapy prediction indicated that higher expression levels correlated with a better response to anti-PD-1 and anti-CTLA-4 therapy (Figure 12H–K).

**FIGURE 13:**
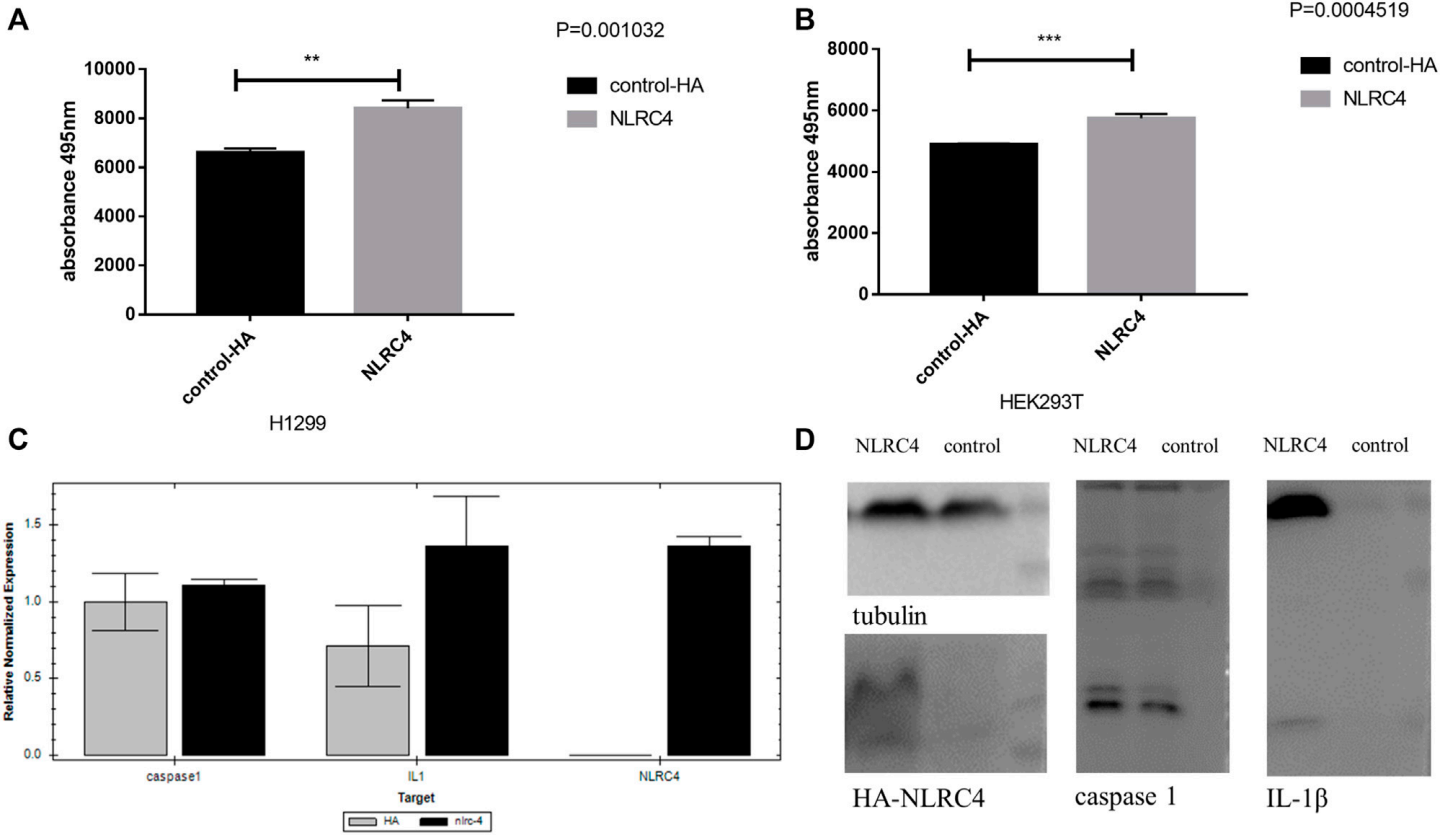
Experimental validation of NLRC4 in pyroptosis and lung adenocarcinoma. **(A,B)** LDH assay. NLRC4 was overexpressed in H1299 and HEK293T cells. We collected the supernatant in each group to test the level of LDH at the absorbance of 495 nm. **(C)** Comparison of mRNA levels of downstream related genes when NLRC4 was overexpressed, the difference of caspase 1 was not very obvious, but a higher level of IL-1β in NLRC4 overexpression group. **(D)** Activation of inflammasome in HEK293T cells by overexpressing NLRC4, pro-caspase 1, ASC and pro-IL-1β. We found that NLRC4 responded to the expression and maturation of caspase-1 and IL-1β. (*t*-test, *p* values were as follows: ^**^ *p* < 0.01; ^***^ *p* < 0.001).

An inflammasome activation system was established in HEK293T cells to study further the probable mechanism linking NLRC4, pyroptosis, and LUAD. We found that overexpressing NLRC4, caspase-1, and IL-1 in HEK293T cells enhanced the maturation of caspase-1 and IL-1. As shown in Figure 13, cleaved IL-1, caspase 1, and LDH levels increased in HEK293T and H1299 cells when NLRC4 was overexpressed, implying the death of more cancer cells (Figure 13).

## Discussion

As an alternative mode of programmed cell death, necroptosis can elicit strong adaptive immune responses that may defend against tumor progression (Salomon, et al., 2018). Necrosis-induced inflammation facilitates tissue repair responses (which are largely immunoregulatory) but not effective anticancer immunity (Tang, et al., 2020). Activation of the canonical programmed necrosis includes the formation of a complex containing receptor-interacting protein kinase-1 (RIP1), RIP3 and recruitment of mixed lineage kinase domain-like protein (MLKL), leading to lytic cell death accompanied by *de novo* production of proinflammatory mediators (Snyder, et al., 2019). Another key mediator in necroptosis includes cellular inhibitor of apoptosis protein 1 (cIAP1/2), deubiquitinase cylindromatosis (CYLD), and caspase-8. CYLD deubiquitinates RPK1 and subsequently limits the sustained activation of NF-κB signaling, while cIAP1/2 polyubiquitinates RIPK1 to induce it (Gong, et al., 2019). Although previous studies pointed out its antitumor immunogenicity through CD8^+^ T cells, recent research proved that immune-mediated tumor control by necroptotic fibroblasts requires nuclear factor κB (NF-κB) activation within dying cells but not MLKL-mediated and cell lysis-dependent DAMP release (Snyder, et al., 2019). And the role of NF-κB activation in necroptosis-provoked antitumor immunity is controversial (Yatim, et al., 2015). MLKL translocation to the plasma membrane is triggered by RIPK3-mediated phosphorylation of MLKL, which results in membrane damage. Consequently, potassium ion efflux may further activate NLRP3 *via* NEK7, which may constitute a cross-talk with the pyroptosis pathway (Tang, et al., 2020). Pyroptosis is a new nonapoptotic form of programmed cell death strongly associated with the inflammatory response by triggering the production of cytokines, such as IL-1 and IL-18, playing a crucial role in modulating the progression of cancer (Hsu, et al., 2021). We developed a PRGs model to analyze the effect of pyroptosis on LUAD progression and possible biochemical pathways to address the potential of integrating these two modalities comprehensively. We also generated a landscape of the differences in the LUAD microenvironment based on these gene signatures to develop personalized anti-cancer therapeutic strategies. In this study, the mRNA levels of 48 PRGs were measured in LUAD and normal tissues, thus obtaining DEGs. We constructed a pyroptosis-related gene model to identify two pyroptosis patterns distinguished by different biological processes and immunological features. When the risk model was applied in a clinical setting, the score accurately predicted the prognosis of individual patients with LUAD. Patients with high scores typically had shorter survival times. The pyroptosis-related score demonstrated substantial correlations with PD-L1, CTLA-4, and immunophenotype, confirming the ability of the risk model to predict the immunotherapy response. In addition, patients with different scores had varying sensitivity to target therapy or chemotherapy, thus providing some suggestions for individualized anti-cancer therapies. Overall, this study showed how pyroptosis influenced the microenvironment in LUAD and highlighted its value in predicting the response to anti-cancer treatment.

Inflammation by innate immune cells designed to fight infections and heal wounds can contribute to the initiation and progression of cancer by secreting growth factors and reactive oxygen species, which induce genomic alterations, chronic and uncontrolled inflammation, and proliferation of malignant cells (Hanahan and Weinberg, 2011). Also, cytokines, chemokines, and a few other substances may enhance proliferation, prevent cell death, and facilitate the migration of cancer through their influence on the TME (Grivennikov, et al., 2010). The inflammasome is a cytosolic immunological signaling complex that causes inflammation and pyroptosis. It is composed of a sensor receptor and an adaptor protein (apoptosis-associated speck-like protein containing a caspase activation and recruitment domains complex). A functional inflammasome is initiated by pattern recognition receptors that can detect pathogen-associated molecular patterns, danger-associated molecular patterns, and homeostasis-altering molecular processes, including nucleotide-binding domain-like receptors (NLRs), absent in melanoma 2-like (AIM2) receptors, and the newly identified pyrin domain–containing receptors (Xue, et al., 2019). Multiple inflammasomes, including NLRP3, NLRC4, NLRP1, and AIM2, may inhibit tumor initiation by influencing innate and adaptive immunity, apoptosis, and differentiation (Di Virgilio, 2013). A previous study discovered that NLRP1, NLRP3, NLRC4, and AIM2 inflammasome complex proteins had pro- or antitumoral properties, especially in breast cancer (Jin and Kim, 2020).

Human NLRP1 was identified as the first protein capable of forming an inflammasome complex. Recent studies indicated that NLRP1 expression was higher in primary breast cancer tissue than in adjacent noncancerous tissue and was associated with lymph node metastasis, tumor-node-metastasis (TNM) stage, and Ki-67. Moreover, NLRP1 enhanced breast cancer cell proliferation, migration, and invasion by inducing epithelial–mesenchymal transition (EMT) (Wei, et al., 2017). The N-terminal oligomerization domain (NOD) proteins, NOD1 and NOD2, are members of the intracellular NOD-like receptor family, which can induce proinflammatory responses. NOD1 was found to be constitutively expressed in epithelial cells, helping in monitoring cytosol integrity and avoiding malignant transformation (Shin, et al., 2018). The overexpression of NOD1 significantly inhibited carcinogenesis *in vivo* and increased the sensitivity of hepatocellular carcinoma cells to chemotherapy *via* blocking the mitogen-activated protein kinase (MAPK) pathway (Ma, et al., 2020).

As with NLRC4, the inflammasome regulated the expression of adipocyte-mediated vascular endothelial growth factor A and angiogenesis, which accelerates breast cancer progression (Kolb, et al., 2016). A previous study showed that NAIP could form an inflammasome with NLRC4, which was related to protection against colitis-associated cancer (Allam, et al., 2015). It was also found to inhibit the hyperactivation of the transcription factor STAT3 as well as the generation of anti-apoptotic and proliferation-related enzymes (Allam, et al., 2015). NLRC4 was demonstrated to be essential for cytokine and chemokine production in macrophages associated with tumors, as well as the generation of IFN-producing CD4^+^ and CD8^+^ T cells that reduced the growth of melanoma tumors in mice (Janowski, et al., 2016). In the 293T inflammatory experiment, we discovered that NLRC4 contributed to the cleavage of pro-IL-1, resulting in pyroptosis. The activation of pro-caspase-1 was responsible for the cleavage of pro-IL-1 and pro-IL-18 proteins into mature active forms and the generation of cytokines in response to pathogen-associated molecular patterns and damage-associated molecular patterns (Rathinam and Fitzgerald, 2016). Moreover, the K-M survival curve showed that a higher level of NLRC4 expression in LUAD was associated with a more favorable prognosis.

And from the risk model, we found that all of these factors were up-expressed in low-risk group, particularly, the level of NLRC4 in lung cancer was also significantly lower in the tumor sample. To investigate its value as a prognostic factor, we compared the survival differences in the two risk groups divided by our model. In clinical practice, we observed that the risk score was an independent factor related to prognosis, with a correlation between increased risk and a worse prognosis, as measured by OS and PFS. It was also effective when we applied it to patients with different stages; high-risk patients had a low probability of survival in both early and advanced stages. Patients with a low-risk score had significantly longer longevity, suggesting that those with a high-risk score should receive more frequent clinical surveillance and appropriate treatment to avoid disease recurrence and progression.

As we discussed above, pyroptosis can trigger crosstalk between innate and adaptive immunity, modulating the cancer microenvironment to induce an immunostimulatory response (Hsu, et al., 2021). As the previous study demonstrated that the context of TME was critical to tumor development and treatment (Thorsson, et al., 2018). Our risk model had numerous similarities with a recent study in which the high-risk group displayed an immune desert and a reduced degree of immune checkpoint expression. Consistent with previous findings, the low-risk group possessed a highly active immune status, including cytokine production, immune receptor activation, and the phosphatidyl-inositol 3-kinase/serine-threonine kinase (PI3K-Akt) signaling pathway, all of which corresponded to a hot tumor phenotype. In the high-risk group, the number of essential anti-tumor-infiltrating immune cells was low, indicating an overall decrease in immune activity. Moreover, the immune microenvironment analyses indicated a global enhancement of immune cell infiltration as well as immune score in low-risk group. These findings indicated that the low-risk group might have a better immune environment.

However, whether it meant a better response to immunotherapy needed further clinical investigation. Since not all patients benefit from immunotherapy, a considerable amount of research has been devoted to the selection of the potentially beneficial population for immunotherapy. From the mechanism of immunotherapy, we can see that T cells are the soldiers of the immune response, while the activation of it requires two kinds of signals: TCR engagement with the MHC–peptide antigen complex (MHC-Ag) on an APC or a target cell, and interaction of the costimulatory receptor CD28 on the T cell with costimulatory B7 molecules (CD80/CD86). However, in response to T-cell activation, the immune checkpoints CTLA4 and PD-1 are upregulated on the T cell and bind to B7 and PD-L1/L2, respectively, to inhibit T-cell activation (Sharma, et al., 2021). Thus, PD-L1 expression is associated with an increased likelihood of response to PD-1 pathway blockade, but responses to ICIs can also be seen in patients with no tumor PD-L1 expression. Moreover, a minority of somatic mutations in tumor DNA can give rise to neoantigens, mutation-derived antigens that are recognized and targeted by the immune system. And TMB can represent a useful estimation of tumor neo-antigenic load, evolving as a relevant tool for the identification of patients likely to respond to immunotherapy (Chan, et al., 2019). Recent investigations pointed out that TMB failed to show predictive accuracy for ICIs response due to a lack of broad ICIs approval. And based on these, we wanted to find if these biomarkers could be improved combined with our risk model. According to the model, we found that patients in different risk groups showed significantly different characteristics, both in terms of TMB and immune checkpoints. The TMB was relatively high in the high-risk group, while the expression of their immune checkpoints was generally low. And the expression of TP53 was relatively higher in the high-risk group. Combined with the risk model, we find that high-risk level with lower TMB has a significantly worst survival probability, which might be an amplification effect of these two parameters. Moreover, the mutation rate of KRAS was also higher in patients with high-risk. Previous research showed that the mutation of KRAS correlated with a low response rate to gefitinib in LUAD, which was consistent with the result of drug sensitivity based on the risk model. For the potential relationship among pyroptosis, TMB and anti-cancer immunity, we need further experimental validation, but for its application in prognosis, it works well. As for these immune checkpoints, take PD-L1 for example, the high-risk group showed relatively low expression. However, from the analysis, we cannot judge the intrinsic mechanism, as the analyses of ESTIMATE showed there were more stromal components and less tumor cells. We assumed that LUAD with higher expression of particular PRGs (genes constructed the risk model) may have a higher infiltration of immune cells, which stimulated the expression of immune related markers, including PD-L1, CD80, LAG3, and so on. The expression of immune checkpoints differed significantly between the high- and low-risk groups, indicating that patients in the low-risk group may benefit more from immune checkpoint inhibitors. However, it is not simply a cause-and-effect relationship, but a mixture with predictive value. As the risk model showed, all of these PRGs’ coefficients were negative, and we could not simply conclude whether the cancer immunity was induced by pyroptosis, or the pyroptosis was facilitated by anti-cancer immunity. For example, paclitaxel, a microtubule-stabilizing agent used in cancer therapy, has been demonstrated to enhance innate immunity by activating the NLRP3 inflammasome in macrophages (Zeng, et al., 2019). From the mechanism we can conclude that cancer immunity is a complex network, while the activation of T cells may be one of the key points. The expression of immune checkpoints showed significant differences between the high and low risk groups, suggesting the potential relationship between pyroptosis and cancer immunity, especially the function and activation of T cells. However, the result from TIDE reflected the profiles at the late stage of T cell dysfunction. The higher score in low-risk group indicated the signatures of tumor immune evasion, which means a worse response to immunotherapy. This has been contradicted by the expression level of PD-L1. As we have discussed, there was no perfect biomarker for predicting the efficacy of immunotherapy. A previous study revealed that immunophenoscore claimed to have good ICIs response in melanoma but worse in TIDE. The reliable TIDE signatures were computed in five cancer types without lung cancer, and only melanoma has publicly available data on tumor expression and clinical outcome of patients treated with anti-PD1 or anti-CTLA4. The mouse tumor models revealed two stages of T cell dysfunction; anti-PD1 treatment can revive the early-stage dysfunctional T cells, and the late-stage dysfunctional T cells are resistant to ICIs reprogramming. Apart from mutation or neo-antigen load, multiple factors could affect immune checkpoint inhibitors’ effectiveness, such as PD-L1 level, degree of cytotoxic T cell infiltration, antigen presentation defects, interferon signaling, mismatch repair deficiency, tumor aneuploidy, intestinal microbiota, and so on. A previous study revealed that uncontrolled activation of the PI3K-Akt pathway at the cellular level might create an immunologically tolerant TME and alter the response to ICIs (Giannone, et al., 2020). Other biomarker types can also predict T cell infiltration and ICIs response, it might achieve higher predictive performance if the risk model could be applied jointly with them.

In addition, the result of TMB revealed a substantial correlation between risk score and TMB. The combination of risk score and TMB could be used as a tool for prognostic stratification. Our study found a higher rate of mutations in high-risk patients, including TP53 and KRAS, suggesting that patients with high-risk scores may activate potentially oncogenic pathways that promote tumor initiation and proliferation. And we also evaluated the chemotherapy as well as target therapy. From the gene mutation, we found that some oncogenes mutation rates were different in the two risk groups. The drug sensitivity revealed that the sensitivity to chemotherapy and targeted therapy differed in different risk groups. Low-risk patients were more sensitive to gefitinib and crizotinib than erlotinib, gemcitabine, docetaxel, and paclitaxel.

To better understand the potential mechanism of pyroptosis in LUAD, we chose NLRC4 for validation. We discovered that its expression in the survival database was negatively connected with survival, corresponding with the negative coefficient in our construction model. NLRC4 is expressed in immune and non-immune cells, including monocytes, macrophages, and neutrophils; nevertheless, differential expression of NLRC4 has been reported in many types of tumor tissue. Studies have shown normal levels in lung cancers, but our findings based on TCGA reveal that it was significantly lower in LUAD and that its expression was associated with prognosis. And our experimental data from H1299 (lung adenocarcinoma cell line) showed that overexpression of NLRC4 could promote pyroptosis by measuring LDH released from dead cells. And the inflammasome activation assay initially validated that NLRC4 could promote IL-1β maturation, which leads to pyroptosis of lung cancer. IL-1β is a key pro-inflammatory cytokine that regulates the expression of several genes involved in the inflammatory process. Besides, previous studies showed that many clinical drugs stimulated and modulated pyroptotic pathways to inhibit tumor growth. Erlotinib decreased the phosphorylation of extracellular signal-regulated kinase (ERK) 1/2 through the PI3K–Akt signaling pathway after lipopolysaccharide treatment and downregulated the expression of TLR4 on macrophages, thereby regulating the microenvironment or systemic anti-tumor immunity (Xue, et al., 2021). Animal models of colorectal cancer have shown that Nlrc4^−/−^ mice displayed increased tumor formation, reduced apoptosis in tumors, and increased proliferation of colonic epithelial cells during the early-stage (Kay, et al., 2020). Consequently, comprehensive studies of pyroptosis and the characteristics of TME in each patient could help us identify the tumor immunological characteristic and guide a more accurate treatment strategy.

Despite providing some novel insights into the immune-oncology correlations of pyroptosis in LUAD, our study had several limitations. First, our pyroptosis signature was derived from public datasets; however, its prognostic value in patients with LUAD receiving immunotherapy requires more validation. Second, the TIDE score was significantly higher in the low-risk group, indicating a poor immunotherapy response based on a previous study. Also, it appeared to be contradictory with other results, such as the PD-L1 expression and the immune score. We wondered whether TIDE or immune checkpoints alone could accurately predict the immunological efficacy of LUAD. The TIDE score combined T cell dysfunction and elimination characteristics to simulate tumor immune escape with varying proportions of tumor-infiltrating cytotoxic T cells. However, abnormalities in antigen presentation, interferon signaling, and mismatch repair can compromise the efficacy of immune checkpoint inhibition therapy, and hence combining them with other immune cells or factors may be preferable.

## Conclusion

The current understanding of pyroptosis, particularly its mechanism in LUAD, is limited. In this study, we investigated the predictive value of PRGs in LUAD. Numerous PRGs were differentially expressed in normal and LUAD tissues, showing a direct correlation between pyroptosis and LUAD. Moreover, the risk score derived from our risk signature was identified as an independent risk factor for LUAD prognosis. The pyroptosis-related risk model outlined the crosstalk and regulatory roles in tumor immunity, as well as their application in cancer treatment. Our model might help in developing personalized cancer treatments for patients with LUAD.

## Data Availability

Publicly available datasets were analyzed in this study. This data can be found here: https://portal.gdc.cancer.gov.

## References

[B1] Allam R. Maillard M. H. Tardivel A. Chennupati V. Bega H. Yu C. W. (2015). Epithelial NAIPs protect against colonic tumorigenesis. J. Exp. Med. 212, 369–383. 10.1084/jem.20140474 25732303 PMC4354369

[B2] Bray F. Ferlay J. Soerjomataram I. Siegel R. L. Torre L. A. Jemal A. (2018). Global cancer statistics 2018: GLOBOCAN estimates of incidence and mortality worldwide for 36 cancers in 185 countries. Ca. Cancer J. Clin. 68, 394–424. 10.3322/caac.21492 30207593

[B3] Chan T. A. Yarchoan M. Jaffee E. Swanton C. Quezada S. A. Stenzinger A. (2019). Development of tumor mutation burden as an immunotherapy biomarker: Utility for the oncology clinic. Ann. Oncol. 30, 44–56. 10.1093/annonc/mdy495 30395155 PMC6336005

[B4] Charoentong P. Finotello F. Angelova M. Mayer C. Efremova M. Rieder D. (2017). Pan-cancer immunogenomic analyses reveal genotype-immunophenotype relationships and predictors of response to checkpoint blockade. Cell Rep. 18, 248–262. 10.1016/j.celrep.2016.12.019 28052254

[B5] Di Virgilio F. (2013). The therapeutic potential of modifying inflammasomes and NOD-like receptors. Pharmacol. Rev. 65, 872–905. 10.1124/pr.112.006171 23592611

[B6] Duma N. Santana-Davila R. Molina J. R. (2019). Non-small cell lung cancer: Epidemiology, screening, diagnosis, and treatment. Mayo Clin. Proc. 94, 1623–1640. 10.1016/j.mayocp.2019.01.013 31378236

[B7] Fang Y. Tian S. Pan Y. Li W. Wang Q. Tang Y. (2020). Pyroptosis: A new frontier in cancer. Biomed. Pharmacother. 121, 109595. 10.1016/j.biopha.2019.109595 31710896

[B8] Fernandes-Alnemri T. Wu J. Yu J. W. Datta P. Miller B. Jankowski W. (2007). The pyroptosome: A supramolecular assembly of ASC dimers mediating inflammatory cell death via caspase-1 activation. Cell Death Differ. 14, 1590–1604. 10.1038/sj.cdd.4402194 17599095 PMC3345951

[B9] Giannone G. Ghisoni E. Genta S. Scotto G. Tuninetti V. Turinetto M. (2020). Immuno-metabolism and microenvironment in cancer: Key players for immunotherapy. Int. J. Mol. Sci. 21, E4414. 10.3390/ijms21124414 PMC7352562 32575899

[B10] Gong Y. Fan Z. Luo G. Yang C. Huang Q. Fan K. (2019). The role of necroptosis in cancer biology and therapy. Mol. Cancer 18, 100. 10.1186/s12943-019-1029-8 31122251 PMC6532150

[B11] Greathouse K. L. White J. R. Vargas A. J. Bliskovsky V. V. Beck J. A. Von Muhlinen N. (2018). Interaction between the microbiome and TP53 in human lung cancer. Genome Biol. 19, 123. 10.1186/s13059-018-1501-6 30143034 PMC6109311

[B12] Grivennikov S. I. Greten F. R. Karin M. (2010). Immunity, inflammation, and cancer. Cell 140, 883–899. 10.1016/j.cell.2010.01.025 20303878 PMC2866629

[B13] Hanahan D. Weinberg R. A. (2011). Hallmarks of cancer: The next generation. Cell 144, 646–674. 10.1016/j.cell.2011.02.013 21376230

[B14] Hirsch F. R. Scagliotti G. V. Mulshine J. L. Kwon R. Curran W. J. Jr. Wu Y. L. (2017). Lung cancer: Current therapies and new targeted treatments. Lancet 389, 299–311. 10.1016/S0140-6736(16)30958-8 27574741

[B15] Hsu S. K. Li C. Y. Lin I. L. Syue W. J. Chen Y. F. Cheng K. C. (2021). Inflammation-related pyroptosis, a novel programmed cell death pathway, and its crosstalk with immune therapy in cancer treatment. Theranostics 11, 8813–8835. 10.7150/thno.62521 34522213 PMC8419056

[B16] Janowski A. M. Colegio O. R. Hornick E. E. Mcniff J. M. Martin M. D. Badovinac V. P. (2016). NLRC4 suppresses melanoma tumor progression independently of inflammasome activation. J. Clin. Invest.. 126, 3917–3928. 10.1172/JCI86953 27617861 PMC5096827

[B17] Jiang P. Gu S. Pan D. Fu J. Sahu A. Hu X. (2018). Signatures of T cell dysfunction and exclusion predict cancer immunotherapy response. Nat. Med. 24, 1550–1558. 10.1038/s41591-018-0136-1 30127393 PMC6487502

[B18] Jin H. Kim H. J. (2020). NLRC4, ASC and caspase-1 are inflammasome components that are mediated by P2Y2R activation in breast cancer cells. Int. J. Mol. Sci. 21, E3337. 10.3390/ijms21093337 PMC7246622 32397236

[B19] Kay C. Wang R. Kirkby M. Man S. M. (2020). Molecular mechanisms activating the NAIP-NLRC4 inflammasome: Implications in infectious disease, autoinflammation, and cancer. Immunol. Rev. 297, 67–82. 10.1111/imr.12906 32729154

[B20] Kolb R. Phan L. Borcherding N. Liu Y. Yuan F. Janowski A. M. (2016). Obesity-associated NLRC4 inflammasome activation drives breast cancer progression. Nat. Commun. 7, 13007. 10.1038/ncomms13007 27708283 PMC5059727

[B21] Ma X. Qiu Y. Zhu L. Zhao Y. Lin Y. Ma D. (2020). NOD1 inhibits proliferation and enhances response to chemotherapy via suppressing SRC-MAPK pathway in hepatocellular carcinoma. J. Mol. Med. 98, 221–232. 10.1007/s00109-019-01868-9 31872284

[B22] Pinto A. Morello S. Sorrentino R. (2011). Lung cancer and Toll-like receptors. Cancer Immunol. Immunother. 60, 1211–1220. 10.1007/s00262-011-1057-8 21789594 PMC11029286

[B23] Rathinam V. A. Fitzgerald K. A. (2016). Inflammasome complexes: Emerging mechanisms and effector functions. Cell 165, 792–800. 10.1016/j.cell.2016.03.046 27153493 PMC5503689

[B24] Reck M. Carbone D. P. Garassino M. Barlesi F. (2021). Targeting KRAS in non-small-cell lung cancer: Recent progress and new approaches. Ann. Oncol. 32, 1101–1110. 10.1016/j.annonc.2021.06.001 34089836

[B25] Salomon B. L. Leclerc M. Tosello J. Ronin E. Piaggio E. Cohen J. L. (2018). Tumor necrosis factor alpha and regulatory T cells in oncoimmunology. Front. Immunol. 9, 444. 10.3389/fimmu.2018.00444 29593717 PMC5857565

[B26] Sharma P. Siddiqui B. A. Anandhan S. Yadav S. S. Subudhi S. K. Gao J. (2021). The next decade of immune checkpoint therapy. Cancer Discov. 11, 838–857. 10.1158/2159-8290.CD-20-1680 33811120

[B27] Shin W. G. Park B. J. Lee S. J. Kim J. G. (2018). Infection of human intestinal epithelial cells by invasive bacteria activates NF-κB and increases ICAM-1 expression through NOD1. Korean J. Intern. Med. 33, 81–90. 10.3904/kjim.2015.409 28092699 PMC5768537

[B28] Snyder A. G. Hubbard N. W. Messmer M. N. Kofman S. B. Hagan C. E. Orozco S. L. (2019). Intratumoral activation of the necroptotic pathway components RIPK1 and RIPK3 potentiates antitumor immunity. Sci. Immunol. 4, eaaw2004. 10.1126/sciimmunol.aaw2004 31227597 PMC6831211

[B29] Tang R. Xu J. Zhang B. Liu J. Liang C. Hua J. (2020). Ferroptosis, necroptosis, and pyroptosis in anticancer immunity. J. Hematol. Oncol. 13, 110. 10.1186/s13045-020-00946-7 32778143 PMC7418434

[B30] Thai A. A. Solomon B. J. Sequist L. V. Gainor J. F. Heist R. S. (2021). Lung cancer. Lancet 398, 535–554. 10.1016/S0140-6736(21)00312-3 34273294

[B31] Thorsson V. Gibbs D. L. Brown S. D. Wolf D. Bortone D. S. Ou Yang T. H. (2018). The immune landscape of cancer. Immunity 48, 812–830. 10.1016/j.immuni.2018.03.023 29628290 PMC5982584

[B32] Wang X. Lin W. Liu T. Xu Z. Wang Z. Cao Z. (2021). Cross-talk of pyroptosis and tumor immune landscape in lung adenocarcinoma. Transl. Lung Cancer Res. 10, 4423–4444. 10.21037/tlcr-21-715 35070752 PMC8743509

[B33] Wei J. Xu Z. Chen X. Wang X. Zeng S. Qian L. (2020). Overexpression of GSDMC is a prognostic factor for predicting a poor outcome in lung adenocarcinoma. Mol. Med. Rep. 21, 360–370. 10.3892/mmr.2019.10837 31939622 PMC6896373

[B34] Wei Y. Huang H. Qiu Z. Li H. Tan J. Ren G. (2017). NLRP1 overexpression is correlated with the tumorigenesis and proliferation of human breast tumor. Biomed. Res. Int. 2017, 4938473. 10.1155/2017/4938473 29214170 PMC5682047

[B35] Wu Z. Man S. Sun R. Li Z. Wu Y. Zuo D. (2020). Recent advances and challenges of immune checkpoint inhibitors in immunotherapy of non-small cell lung cancer. Int. Immunopharmacol. 85, 106613. 10.1016/j.intimp.2020.106613 32450531

[B36] Xue Q. Liu X. Chen C. Zhang X. Xie P. Liu Y. (2021). Erlotinib protests against LPS-induced parthanatos through inhibiting macrophage surface TLR4 expression. Cell Death Discov. 7, 181. 10.1038/s41420-021-00571-4 34282120 PMC8290014

[B37] Xue Y. Enosi Tuipulotu D. Tan W. H. Kay C. Man S. M. (2019). Emerging activators and regulators of inflammasomes and pyroptosis. Trends Immunol. 40, 1035–1052. 10.1016/j.it.2019.09.005 31662274

[B38] Yatim N. Jusforgues-Saklani H. Orozco S. Schulz O. Barreira Da Silva R. Reis E Sousa C. (2015). RIPK1 and NF-κB signaling in dying cells determines cross-priming of CD8⁺ T cells. Science 350, 328–334. 10.1126/science.aad0395 26405229 PMC4651449

[B39] Zeng Q. Z. Yang F. Li C. G. Xu L. H. He X. H. Mai F. Y. (2019). Paclitaxel enhances the innate immunity by promoting NLRP3 inflammasome activation in macrophages. Front. Immunol. 10, 72. 10.3389/fimmu.2019.00072 30761140 PMC6361797

[B40] Zhang C. C. Li C. G. Wang Y. F. Xu L. H. He X. H. Zeng Q. Z. (2019). Chemotherapeutic paclitaxel and cisplatin differentially induce pyroptosis in A549 lung cancer cells via caspase-3/GSDME activation. Apoptosis 24, 312–325. 10.1007/s10495-019-01515-1 30710195

[B41] Zhang J. Zhang Q. Lou Y. Fu Q. Chen Q. Wei T. (2018). Hypoxia-inducible factor-1α/interleukin-1β signaling enhances hepatoma epithelial-mesenchymal transition through macrophages in a hypoxic-inflammatory microenvironment. Hepatology 67, 1872–1889. 10.1002/hep.29681 29171040

